# Semiconducting Carbon Nanotubes with Light‐Driven Gating Behaviors in Phototransistor Memory Utilizing an N‐Type Conjugated Polymer Sorting

**DOI:** 10.1002/smsc.202300268

**Published:** 2024-02-22

**Authors:** Yi‐Hsuan Tung, Shang‐Wen Su, En‐Jia Su, Guo‐Hao Jiang, Chun‐Chi Chen, Sheng‐Sheng Yu, Chi‐Cheng Chiu, Chien‐Chung Shih, Yan‐Cheng Lin

**Affiliations:** ^1^ Department of Chemical Engineering National Cheng Kung University Tainan 70101 Taiwan; ^2^ Department of Chemical Engineering and Materials Engineering National Yunlin University of Science and Technology Yunlin 64002 Taiwan; ^3^ Advanced Research Center for Green Materials Science and Technology National Taiwan University Taipei 10617 Taiwan

**Keywords:** conjugated polymers, field‐effect transistors, naphthalene diimide, photomemories, single‐walled carbon nanotubes

## Abstract

Semiconducting single‐walled carbon nanotubes (*s*‐SWNTs) have arisen a growing interest in field‐effect transistors (FETs) due to their advantages, such as lower fabrication temperature, flexibility, and solution processing applicability, compared to traditional silicon‐based FETs. In this study, diversifying the functionality of *s*‐SWNT‐based FETs is focused on, particularly emphasizing their use in nonvolatile photomemory applications. By selectively wrapping *s*‐SWNT with n‐type conjugated polymers (CPs), electron‐trapping and photoresponsive capabilities are endowed in the device. After optimizing the structure and aggregating behavior of n‐type CPs, a favorable supramolecular network comprising *s*‐SWNT and CPs is formed and applied in phototransistor memory. Accordingly, the device exhibits a high memory ratio and window of 10^5^ and 75.7 V, representing its remarkable charge‐storage capabilities. In addition, the device demonstrates decent long‐term stability over 10^4^ s and multilevel memory behavior driven by the varied gate bias or accumulated light‐gating periods. The proposed memory mechanism involves electrical writing and photoerasing processes with the existence of n‐type CPs on *s*‐SWNT, revealing the underlying principles of charge transfer between their heterojunction interfaces. Herein, this research contributes to developing advanced phototransistor memory, offering a promising avenue for future electronic applications.

## Graphical abstract

A series of naphthalene‐diimide‐based conjugated polymers are developed and applied in the sorting process of single‐walled carbon nanotubes to trigger the photoinduced gating behavior in transistor memory.

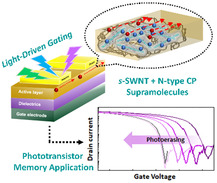

## 1 Introduction

Organic field‐effect transistors (FETs) have recently received considerable attention due to their lower fabrication temperature and cost than traditional silicon‐based transistors. Unlike conventional semiconductors, conjugated polymers (CPs) served as organic semiconductors possess several advantages, such as large‐area flexibility, solution processability, and suitability for stretchable electronic applications.^[^ ^1^, ^2^, ^3^ ^]^ Within the past decade, many research groups have dedicated their efforts to increasing CPs’ magnitude of mobility from below 0.01 to beyond 1 cm^2^ V^−1^ s^−1^.^[^ ^4^ ^]^ With the advantages of nondestructive readout, compatibility with integrated circuits, and solution processability, diversified organic materials have contributed to a rising trend in the development of nonvolatile memory.^[^ ^5^ ^]^ By suitably endowing charge storage and slow polarization of the gate bias through chemical structure or device architecture engineering, organic FET is capable of transforming into nonvolatile memory. Typically, organic FET employs three primary strategies to create interfacial charge traps to induce memory effects: 1) incorporating intrinsic countercharge traps in the semiconducting channel; 2) integrating the memory effect at the organic semiconductor/dielectric interface; and 3) incorporating a floating gate dielectric or chargeable electret beneath the semiconducting channel.^[^ ^6^, ^7^ ^]^ To date, with rapid advancements, many research studies have suggested that light can also be utilized to entirely or partially replace electrical operation, showing orthogonal working principles.^[^ ^8^, ^9^ ^]^ On the basis of this similar concept, it is possible to induce charge trapping/storing by photoexcitation and replace the application of gate impetus. Therefore, organic phototransistor memory has been extensively developed, showing ultrahigh photoresponsivity, multilevel memory storage, and decent stability.^[^ ^10^, ^11^, ^12^ ^]^ For example, a photoactive polymer electret or floating gate can be introduced into organic FET to trigger photoresponse and memory behavior.^[^ ^13^, ^14^ ^]^ However, phototransistor memory with a bilayered structure may be confined by the distribution of the floating gate nanoparticles or intrinsic carrier traps of the polymer electret. The instability and incompatibility between the channel and the electret layer limit the photomemory performances.^[^ ^15^ ^]^ Some research groups have introduced the single‐layered structure where the channel incorporates photogates. Within this type of phototransistor memory, the photogate can serve as a charge‐trapping site. It can effectively improve the separation of excitons due to the heterojunction between the semiconductor and the photogate.^[^ ^16^, ^17^ ^]^

For a single‐layered structure phototransistor, single‐walled carbon nanotubes (SWNTs) are highly suitable as the active layer material due to their high mobility, large surface area, and compatibility with solution‐processing techniques. By employing appropriate chemical doping or forming a bulk heterojunction, photoresponsive behavior can be induced. However, commercially available SWNTs contain a mixture of approximately two‐third semiconducting SWNTs (*s*‐SWNTs), one‐third metallic SWNTs (*m*‐SWNTs), amorphous carbon, and residual catalysts. Thus, purification becomes a significant issue in FET fabrication. Generally, *s*‐SWNTs isolation can be classified into three approaches: 1) density gradient ultracentrifugation;^[^ ^18^, ^19^ ^]^ 2) size‐exclusion chromatography;^[^ ^20^ ^]^ and 3) noncovalent selective sorting of *s*‐SWNTs with CPs.^[^ ^21^, ^22^, ^23^, ^24^ ^]^ The last of these methods, which often employs polyphenylenevinylene‐,^[^ ^21^ ^]^ polyfluorene‐,^[^ ^22^ ^]^ and poly(3‐alkylthiophene)‐based polymers,^[^ ^23^, ^24^ ^]^ is particularly esteemed for its exceptional efficiency in dispersion, selectivity, and cost‐effectiveness. Typically, CPs bound to *s*‐SWNT surfaces are removed to diminish energetic barriers that hinder carrier transport between *s*‐SWNTs, adversely affecting device electrical performance. However, from the perspective of the transistor's operational mechanism, if a narrow‐bandgap CP is used for sorting and achieves proper energy alignment with the source/drain electrodes, the presence of CPs may not significantly impair device performance, even if not removed. Moreover, these bound CPs can act as photosensitive agents; upon light excitation, they can transfer excitons to the *s*‐SWNTs, thereby modulating channel conductivity. This approach has the potential to enhance the performance of contemporary phototransistor memory. Nowadays, organic phototransistor memory still has some issues that need to be solved. For instance, the photoresponsivity is relatively low due to their poor carrier mobility.^[^ ^25^, ^26^ ^]^ This result is unsatisfactory because a low photocurrent may disable the discernibility of memory states and deteriorate their long‐term stability. The selection of polymer materials is crucial, as they directly impact the creation, separation, and transfer of photogenerated excitons, in addition to influencing charge trapping at the interface.^[^ ^27^ ^]^ Striking a balance between enhancing mobility and modulating the heterojunction, which significantly affects the performance of phototransistor memory, still presents a challenge.^[^ ^28^ ^]^ To overcome these issues, *s*‐SWNTs seem to offer a viable solution for surpassing mobility constraints. Furthermore, choosing CPs that can form suitable heterojunctions with *s*‐SWNTs for sorting makes it possible to effectively facilitate the transfer of photogenerated excitons. The interaction between *s*‐SWNTs and the dielectric layer at the interface can generate trapping sites, achieving the desired memory function.

In this study, we report a series of n‐type CPs selectively wrapping *s*‐SWNTs to form a supramolecular network. The n‐type CPs are a series of copolymers consisting of naphthalene diimide (PNDI) and different donor units of thiophene (PNDI‐T), thienothiophene (PNDI‐TT), selenophene (PNDI‐Se), bifluorothiophene (PNDI‐2TF), and bithiophene (PNDI‐2T). The n‐type CPs selectively wrap the medium‐diameter plasma‐discharge (PD) SWNTs. To gain insight into the sorting of *s*‐SWNT with CPs and their supramolecules, optical properties with ultraviolet–visible–near‐infrared (UV–Vis–NIR) absorption and Raman spectroscopy, morphological properties with atomic force microscopy (AFM) were evaluated, and their phototransistor memory characteristics were systematically corroborated. We found that the sorting efficiency of CPs to *s*‐SWNTs is highly related to their molecular interaction revealed by molecular dynamics (MD) simulations and the aggregating behaviors of the CPs to form a supramolecular network with *s*‐SWNTs. The heterojunction between the CPs and *s*‐SWNTs endows with charge‐trapping capability for phototransistor memory application. Previously, *s*‐SWNTs were blended with perovskite nanocrystals or quantum dots for phototransistors in light detection.^[^ ^29^, ^30^ ^]^ The ambient stability will be confined by the formulation of nanocomposites, and the OFF‐state current is too high to be applied in photomemory. Therefore, with this photoactive channel structure, the weak photoresponse and charge‐trapping ability of *s*‐SWNTs may be compensated by n‐type CPs while maintaining the high carrier mobility of *s*‐SWNTs. The dispersed n‐type CPs could serve as a charge‐trapping medium in the *s*‐SWNTs channel to endow the light‐driven gating behavior. Accordingly, the phototransistor memory showed a high memory ratio (*I*_ON_/*I*_OFF_) of 10^5^ and a high memory window (Δ*V*_th_) of 74.4 V. In addition, the device demonstrated multilevel memory behavior that was fulfilled by electrical writing and multiple photoerasing. Finally, a proposed memory mechanism that involves hole‐trapping and light‐driven gating processes was described in this study.

## 2 Results and Discussion

### 2.1 Carbon Nanotube Sorting and Characterization

**Figure 1:**
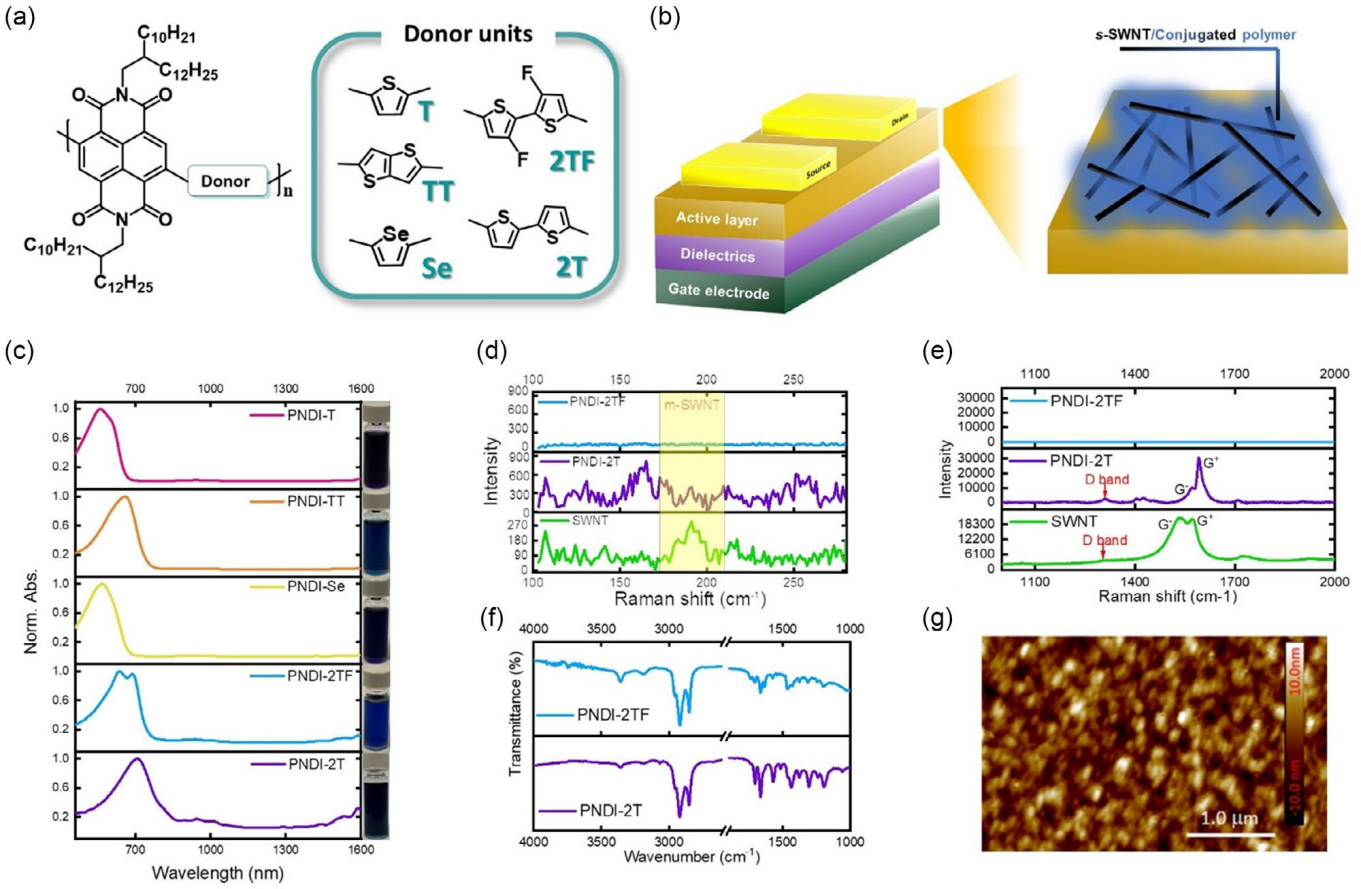
a) Chemical structures of the NDI‐based n‐type CPs. b) The device structure of the phototransistor memory with the channel comprising *s*‐SWNT/n‐type CPs. c) UV–vis absorption spectra of the sorted *s*‐SWNT/n‐type CP solutions. Note that the inset figures on the right‐hand side are the photographs of the sorted solutions. Raman spectra of pristine *s*‐SWNT and *s*‐SWNT sorted by PNDI‐2T or PNDI‐2TF with an excitation wavelength of 633 nm at different Raman shift bands spanning the range of d) 100–300 nm or e) 1000–2000 nm. f) FTIR spectra of the *s*‐SWNT/CPs films comprising PNDI‐2T or PNDI‐2TF. g) AFM topography of the *s*‐SWNT/PNDI‐2T film.

CPs selectively wrapping *s*‐SWNTs through a sorting process have been considered an efficient method for fabricating *s*‐SWNT‐based FETs. In this study, a series of NDI‐based CPs was designed to sort SWNTs selectively. The chemical structures of the CPs are shown in **Figure** **1**a; their syntheses are detailed in Scheme S1 (Supporting Information), and chemical structural characterizations are described in Figure S1, Supporting Information, (size‐exclusion chromatography) and Figure S2–S6, Supporting Information (^1^H nuclear magnetic resonance spectra [NMR]), Supporting Information. In the gel permeation chromatography (GPC) profiles, the peaks in the high‐molecular‐weight range can be derived from the aggregations of polymer chains within the sample, while the peaks in the low‐molecular‐weight range may represent individual chains of CPs. Within these n‐type CPs, different donor units may induce different spacer sizes, electron‐donating abilities, steric hindrances, and chain coplanarities. These properties may have caused the different outcomes in SWNT sorting. The sorting process was accomplished by sonicating a mixture of CP and PD SWNTs using a tip‐sonicator; consequently, the mixture was centrifugated to remove *m*‐SWNTs and impurities. The *s*‐SWNTs adsorption process was conducted by soaking the sorted solution, and the *s*‐SWNT/CP supramolecules could be densely adsorbed onto a hydrophilic substrate.^[^ ^31^ ^]^ Subsequently, poly(methyl methacrylate) (PMMA) was used to transfer the *s*‐SWNT/CP supramolecules to the device substrate. By thermally depositing the top‐contact channel electrodes, the *s*‐SWNT‐based FET was fabricated, as shown in Figure 1b.

The observation of sorting solutions and UV–Vis–NIR optical characterization results are shown in Figure 1c. As can be seen, PNDI‐2T and PNDI‐2TF exhibited more obvious *s*‐SWNT signals than those of PNDI‐T, PNDI‐Se, and PNDI‐TT. The *M*_11_ and *S*_22_ signals of PD *s*‐SWNT range from ≈625 to 1183 nm. To evaluate the sorting efficiency of a CP dispersant, selectivity (*ϕ*) and yield are usually calculated. The purity of SWNTs depends on the *ϕ* value, defined as the integral of *S*_22_ peaks absorbance divided by the integral of *S*_22_ peaks absorbance and the baseline absorbance: *ϕ* = *A*_S22_/(*A*_S22_ + *A*_baseline_).^[^ ^18^ ^]^ The calculation of the *s*‐SWNT yield is evaluated by the UV–vis absorbance using Beer's law, (*λ*)* = εbc*, where *c* represents the concentration of sorting *s*‐SWNTs, and *ε* is evaluated by the UV quantification. In contrast, the yield of *s*‐SWNTs can be calculated by using the following equation: $\text{Yield}=\left(C_{s‐\text{SWNT}}\times V_{\text{sorting}}\right)/\left(\frac{2}{3}W_{\text{SWNT}}\right)$, where *C*_*s*‐SWNT_ and *V*_sorting_ are the concentration and total volume of sorted *s*‐SWNT solutions, and *W*_SWNT_ is the total weight of SWNTs introduced into the sorting process. As mentioned earlier, the commercially available SWNTs contain a mixture of approximately two‐third *s*‐SWNTs, one‐third *m*‐SWNTs, amorphous carbon, and residual catalysts. The *ϕ* values of PNDI‐T, PNDI‐TT, PNDI‐Se, PNDI‐2TF, and PNDI‐2T are 0.36, 0.27, 0.35, 0.21, and 0.36, respectively (Figure S7, Supporting Information). Higher *ϕ* values indicate higher *s*‐SWNT purities, and *ϕ* values > 0.40 were correlated with purity > 99%.^[^ ^32^ ^]^ The yields of PNDI‐T, PNDI‐Se, PNDI‐TT, PNDI‐2TF, and PNDI‐2T are 1.9%, 0.7%, 0.9%, 2.8%, and 18.3%. Although the purities of PNDI‐T and PNDI‐Se are pretty high, their yields are poor. The optimized donor spacer sizes, electron‐donating abilities, and polymer aggregations possibly cause the overwhelming high yield of PNDI‐2T. It will be further discussed in later sections.

### 2.2 Characterization of *s*‐SWNT/CP Supramolecule Films

To further characterize the enrichment of *s*‐SWNTs, Raman spectroscopy of 633 nm laser excitations was applied to test the drop‐cast films. Under this excitation, the strong radial‐breathing‐mode (RBM) peaks of *m*‐SWNTs in the range of 170–210 cm^−1^ (maximal peak at 194 cm^−1^) were observed in the pristine PD SWNTs film. The SWNT diameter can be evaluated by the relation *w*_RBM_ = *A*/d*t* + *B*. For medium‐diameter SWNT, the *A* = 234 cm^−1^ and *B* = 10 cm^−1^. Through this relation, the diameter of PD SWNTs can be approximately evaluated as 1.27 nm. In Figure 1d, the RBM peak is barely visible after the sorting process, indicating that *m*‐SWNTs were removed during the sorting process. In the high‐wavenumber Raman region, the G‐band indicates SWNT features, which comprise two main parts. The G^+^ band is about 1590 cm^−1^, which indicates carbon atom vibrations along the SWNT axis. The G^−^ band is about 1570 cm^−1^, which indicates carbon atom vibrations along the circumferential direction of the SWNT. The lineshape of the G band can also differentiate whether the SWNT is metallic (Breit–Wigner–Fano lineshape) or semiconducting (Lorentzian lineshape).^[^ ^33^ ^]^ Accordingly, the lineshape of pristine SWNT before enrichment in Figure 1f shows the metallic form. However, the PNDI‐2T sorting solution displays a semiconducting form, which indicates that *m*‐SWNTs have been removed during sorting and enrichment. Although the RBM peak of the PNDI‐2TF sorting solution is barely visible within the range of 170–210 cm^−1^, the absence of G band in the Raman spectroscopy suggests that PNDI‐2TF is not an effective dispersant for selectively wrapping PD SWNTs, such as PNDI‐T, PNDI‐TT, and PNDI‐Se (Figure S8, Supporting Information). Furthermore, the ratio of G and D bands can characterize the density of defects, and a higher ratio indicates fewer defects. In Figure 1e, the G/D ratio of *s*‐SWNT/PNDI‐2T can be evaluated as 11.9, indicating its order graphitic structure.

To characterize whether CPs stably wrap on the *s*‐SWNT, Fourier‐transform infrared spectroscopy (FTIR) characterization was applied, as can be seen in Figure 1f and S9 (Supporting Information) for PNDI‐2T/PNDI‐2TF and PNDI‐T, PNDI‐Se, and PNDI‐TT, respectively. In the FTIR spectrum, several important peaks were observed: the peak at 1370 cm^−1^ corresponds to the stretching of C─N in the imide group, while the peak ranges from 1566 to 1650 cm^−1^ represents the stretching of C═C in aromatic rings. In addition, the peak at 1680 cm^−1^ corresponds to the stretching of C═O in tertiary amide, and the peak range from 1650 to 2000 cm^−1^ signifies the bending of C─H in aromatic compounds. Furthermore, the peak ranges from 2850 to 2950 cm^−1^, corresponding to the stretching of C─H in side‐chain alkanes. These peaks provide valuable insights into the composition and structure of CPs in the supramolecules.

The AFM technique was applied to understand the surface morphology of supramolecule films. The AFM topography of *s*‐SWNT/PNDI‐2T is shown in Figure 1g. As can be seen, the surface of the supramolecule film was covered with PNDI‐2T, and the SWNT morphology is slightly visible beneath the PNDI‐2T aggregates. However, the high concentration of supramolecules hinders *s*‐SWNTs morphology on the thin film surface. Herein, the diluted sorting solution can help to get insight into the morphology of SWNTs. The AFM topography using a diluted solution during the film deposition is shown in Figure S10 (Supporting Information). In Figure S10a (Supporting Information), a nanofibrillar structure can be observed, indicating the effective dispersion of *s*‐SWNTs by PNDI‐2T. To extract the details of SWNTs, the contour structure was identified using GTFiber software and the setting parameters are presented in Figure S11 (Supporting Information).^[^ ^34^ ^]^ As can be seen in Figure S10b (Supporting Information), the fibers show an average length of 238 nm. This result reveals the wrapping and accommodating of *s*‐SWNT with PNDI‐2T that embeds *s*‐SWNT in the supramolecule film. With this favorable spatial allocation, the enriched *s*‐SWNT content and heterojunction interfaces between *s*‐SWNT and PNDI‐2T can conduce to improving the charge‐trapping ability and photoresponse in phototransistor memory.

### 2.3 Theoretical Calculations

**Figure 2:**
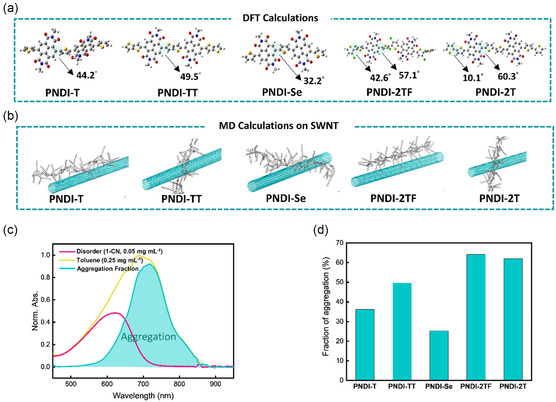
a) Optimized molecular structures of the repeating units in n‐type CPs derived from DFT calculations. b) MD simulation results of interaction between SWNTs and n‐type CPs. c) Deconvolution of the aggregation and disorder fractions in the UV−vis absorption spectra of PNDI‐2T solutions. d) Summary of the aggregation fractions in the toluene solutions of CPs.

To gain a more profound understanding of the structural conformation of CPs that influence the sorting of SWNTs, MD and density‐functional theory (DFT) simulations can help us approach the information of polymer's conformation and their interaction with SWNTs. In this study, DFT calculation was first used to determine the equilibrium state of atoms, providing a comprehensive analysis encompassing bond length, dipole moment, dihedral angles, etc. A particular focus on the dihedral angle emerged as instrumental in elucidating the coplanarity of CPs’ backbones. It is worth noting that two NDI and three donor units were connected to represent the polymer's repeating unit, and their side chains on the NDI unit were replaced with a methyl group to simplify the optimization. As can be seen in **Figure** **2**a and S12, Supporting Information, PNDI‐Se has the smallest dihedral angle between the NDI and donor unit (32.2°). PNDI‐T and PNDI‐TT present higher dihedral angles of 44.2° and 49.5°, respectively. PNDI‐2TF (57.1°) and PNDI‐2T (60.3°) exhibit much larger dihedral angles than other CPs. In addition, the dihedral angles inside the donor units of PNDI‐2TF and PNDI‐2T are 42.6° and 10.1°, respectively. This discrepancy can be attributed to the divergent steric hindrances imposed by hydrogen and fluorene atoms appended to thiophenes, which indicate the strong steric hindrance between the fluorine atoms in PNDI‐2TF. It is worth noting that CPs with a smaller donor unit correlate with a diminished dihedral angle, implying a better chain coplanarity. In contrast, CPs with a larger donor unit indicate poor chain coplanarity and potentially better chain aggregating capability due to the flexible conjugated chains. This concept has been evidenced by Wedler et al. that the more flexible backbone tends to form H aggregation than the planar backbone. Furthermore, the flexibility of the backbone facilitates the establishment of intermolecular contact points.^[^ ^35^ ^]^

Next, MD simulation can provide in‐depth insight into the intricate interaction between *s*‐SWNT/CPs. These five CPs with different donor groups were built with 12 repeats and interacted with a 19.6 nm long (9,9) armchair SWNT (see Experimental Section). Figure 2b directly shows the conformation of CPs attached to the SWNT, and Figure S12 (Supporting Information) indicates the snapshots in the top view to illustrate the *π*–*π* interaction between SWNTs and n‐type CPs. These results demonstrate aggregation dynamics and the nuanced interaction between polymer side chains or backbones with SWNT. Remarkably, the results for PNDI‐2T showcased an adept wrapping of SWNTs by the soft backbone, with minimal obstruction caused by the donor group atoms, thus affirming the sorting capabilities of this specific compound. Next, we observed that the interaction of SWNT with PNDI‐T, PNDI‐TT, and PNDI‐Se were obstructed from approaching the backbones and were held outside the side chains. MD simulations serve to clarify contact conditions and forces between the polymer and SWNT. Moreover, the results of these simulations can be instrumental in corroborating the yields of the sorting process. In the case of PNDI‐2TF, the fluorine atoms have a considerable steric hindrance that blocks the wrapping of SWNTs by CPs. In contrast, PNDI‐2T has adequate steric hindrance and poor coplanarity to make the chains with sufficient surface available to contain the SWNTs. Consequently, PNDI‐2T and PNDI‐2TF yielded 18.3% and 2.8% of *s*‐SWNTs, respectively. In addition, PNDI‐Se has the smallest coplanar angle that leads to poor aggregation, resulting in a low SWNTs yield of only 0.9%.

### 2.4 Aggregating Behaviors of CPs

Aggregation of CPs can be evaluated by UV–vis absorption characterization. This characterization can be helpful in gaining insight into the morphology of CP allocations. The optical absorption spectra are shown in Figure 2c and S13 (Supporting Information) for PNDI‐2T and other CPs, and the summary of CPs’ aggregation fractions is presented in Figure 2d. Note that the disordered state is defined from the polymer solutions of 1‐chloronaphthalene (1‐CN) at 0.05 mg mL^−1^; the polymer solutions in toluene were prepared at a fixed concentration of 0.25 mg mL^−1^, which is the same as in the sorting solution. The calculation of the aggregate fraction was based on the integral of the aggregation and disorder area ratios in the toluene, and the details can be seen in Experimental Section. PNDI‐2TF and PNDI‐2T exhibit higher aggregation fractions of >60%, while the aggregation fractions of PNDI‐T, PNDI‐TT, and PNDI‐Se are 36%, 49%, and 24%, respectively. As can be seen from the DFT simulation and GPCs results, PNDI‐2TF and PNDI‐2T show longer polymer lengths. Choi et al. and Tran et al. previous studies revealed that the longer NDI polymer chains lead to a stronger polymer aggregation.^[^ ^36^, ^37^ ^]^ These two polymers exhibit higher aggregation fractions than the others due to their long‐length and flexible polymer backbones. Despite the comparable polymer chain lengths observed in PNDI‐TT and PNDI‐2T, the diminished aggregation by 10% in PNDI‐TT can be attributed to its inherent chain rigidity as shown in the DFT simulation. PNDI‐TT possesses a smaller dihedral angle (49.5°) than that of PNDI‐2T (60.3°). Therefore, PNDI‐TT exhibits better coplanarity than PNDI‐2T resulting in less aggregation fraction. In contrast, short chains and better backbone coplanarity, such as PNDI‐T and PNDI‐Se, correspond to a lower aggregation ratio. The results indicate that soft backbones with long chain lengths can mitigate the steric hindrance between polymer chains, and the polymers are easily distorted, further causing aggregation and entanglement, thus enhancing the aggregations that can create a reticular structure to wrap SWNTs effectively. However, rigid backbones can hardly be distorted due to their inflexible structure and strong steric hindrance. Even with a long chain length, the inflexibility of the backbone still contributes to the deteriorated aggregations. Furthermore, the result may effectively influence the sorting yields. The strong aggregation of PNDI‐2T exhibits a relatively high sorting yield. However, the yield of PNDI‐2TF is much lower than PNDI‐2T, possibly because of the additional steric hindrance from the fluorine atom on the donor unit, thereby hindering the wrapping on *s*‐SWNTs. The low sorting yields of PNDI‐T, PNDI‐TT, and PNDI‐Se with weak aggregation cannot effectively sort *s*‐SWNTs to form a homogeneous supramolecule network due to their poor aggregating/entanglement ability.

### 2.5 Device Characteristics of the Phototransistor Memory

**Figure 3:**
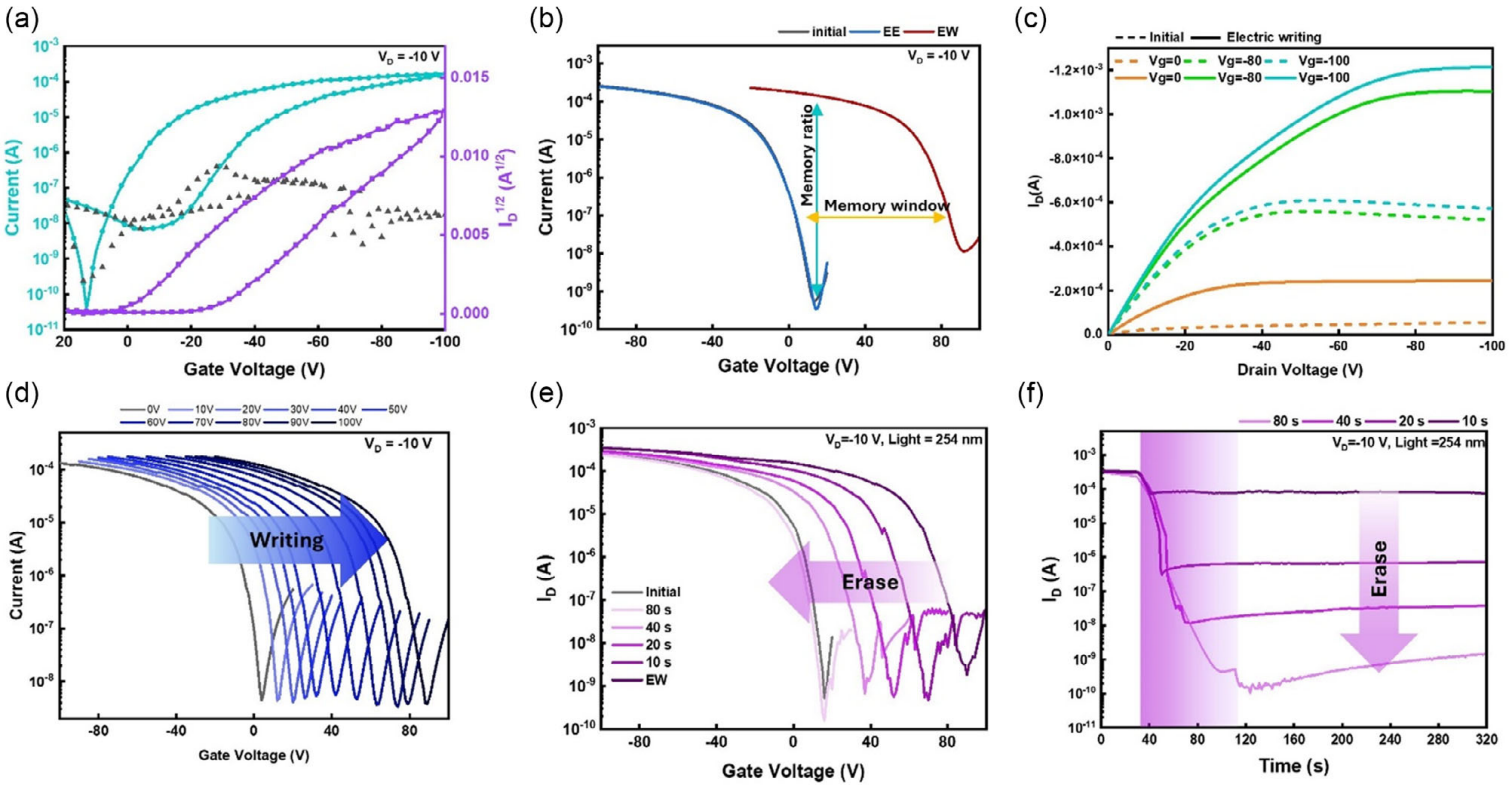
a) Transfer characteristics of the transistor memory. Note that the cyan blue, gray, and purple lines indicate the drain current (*I*_D_), gate current (*I*_G_), and the square root of the drain current (*I*_D_^1/2^). The calculated hole mobility (*μ*_h_) is 0.50 cm^2^ V^−1^ s^−1^. b) Transfer characteristics of the transistor memory at initial (black), electrical writing (EW, *V*_g_ = 100 V for 1 s, red), and electrical erasing (EE, *V*_g_ = −100 V for 1 s, blue) states. c) Output characteristics of the transistor memory at electrical writing (*V*_g_ = 100 V, 1 s, solid), and electrical erasing (*V*_g_ = −100 V, 1 s, dashed) states. d) Transfer characteristics with different electrical writing voltage of *V*_g_ = 10–100 V. e) Transfer and f) transient photocurrent characteristics of the phototransistor memory with light‐driven erasing (254 nm; 0.1 mW cm^−2^) spanning the range of 10–80 s. Note that the measurements were conducted at *V*_d_ = −10 V.

After investigating the morphology of *s*‐SWNT/PNDI‐2T, their FET device performance was next characterized. The *s*‐SWNT/PNDI‐2T supramolecules adsorbed and transferred onto a silicon wafer with 300 nm SiO_2_ covered with a layer of cross‐linked polymer dielectric comprising styrene–butadiene–styrene (SBS) rubber and cross‐linkers. The device fabrication procedure is detailed in Experimental Section, and the bottom‐gate top‐contact (BG/TC) FET device structure is displayed in Figure 1b. Considering the high sorting efficiency of PNDI‐2T, the device channel was fabricated based on the *s*‐SWNT/PNDI‐2T supramolecules. Through the electrical measurement, the FET demonstrated typical p‐type transport properties. The transfer and output characteristics of the phototransistor memory were conducted and presented in **Figure** **3**a–c, S14, and S15 (Supporting Information), and the hole mobility (*μ*_h_), threshold mobility (*V*_th_), memory window (Δ*V*_th_), and memory ratio (*I*_ON_/*I*_OFF_) are summarized in Table S1 (Supporting Information). The transfer characteristic of the reference device comprising pristine *s*‐SWNT is presented in Figure S16, Supporting Information. The reference device was fabricated by using an imine‐linked CP (poly[(9,9‐di‐*n*‐dodecyl‐2,7‐fluorendiyl‐dimethine)‐(1,4‐phenylene‐dinitri‐lomethine)]), which can be depolymerized into monomers and be cleanly removed under mild acidic conditions, yielding polymer‐free *s*‐SWNTs.^[^ ^32^ ^]^ Transfer characteristics of the initial and photoerasing states were recorded by sweeping the gate voltage (*V*_g_) from 20 to –100 V, while the electrical writing state was recorded by sweeping the *V*_g_ from 100 to –20 V. As can be seen in Figure 3a,b, the maximum drain current can reach as high as 10^−3^ A. In our study, the active layer of *s*‐SWNT/PNDI‐2T supramolecules provided promising hole‐transport ability due to the high mobility of SWNTs. Within the calculation, the hole mobility (*μ*_h_) of the FET is high in a value of 0.50 cm^2^ V^−1^ s^−1^ (*V*_d_ = –10 V) and 2.53 cm^2^ V^−1^ s^−1^ (*V*_d_ = –100 V). The charge‐transfer complex between *s*‐SWNT/PNDI‐2T in the supramolecules didn't disrupt the charge transport in the channel. This high carrier‐transport performance outperforms the reported phototransistor memory systems with organic crystalline molecules or CPs as a channel with mobility spanning the range of 0.01–1 cm^2^ V^−1^ s^−1^.^[^ ^11^, ^12^, ^13^, ^14^, ^15^, ^16^, ^17^ ^]^ In Figure S16 (Supporting Information), it can be observed that the *V*_th_ of the device with *s*‐SWNT/PNDI‐2T is significantly more negatively shifted than that of the reference device with *s*‐SWNT (*V*_th_ = 13.7 V). The electron affinity characteristics of PNDI‐2T result in a greater accumulation of hole carriers for conduction than that with pure *s*‐SWNT. Additionally, it is noteworthy that the *μ*_h_ of the device with pure *s*‐SWNT (5.34 cm^2^ V^−1^ s^−1^) is approximately an order of magnitude higher than that of *s*‐SWNT/PNDI‐2T. This disparity indicates the existence of PNDI‐2T in the supramolecules and the different charge affinity between *s*‐SWNT and PNDI‐2T.

Next, the electric writing was conducted by applying *V*_g_ of 100 V for 1 s, and the electrical erasing (*V*_g_ = –100 V, 1 s) or photoerasing was applied at *V*_d_ of –10 V with 254 nm light illumination to switch back the current state. It is worth noting that the concept of photoerasing presents a compelling alternative that has the potential to replace traditional electrical erasing methods. The memory parameters, defined by the individual transfer curves after device programming, were next investigated. The *I*_ON_/*I*_OFF_ and Δ*V*_th_ are evaluated as 10^5^ and 75.7 V at *V*_d _= –10 V (Figure 3b) and 10^3^ and 74.4 V at *V*_d _= –100 V (Figure S15, Supporting Information). Note that the *I*_ON_/*I*_OFF_ was defined at *V*_g_ = –10 V. The shift of the transfer curve can originate from charge traps at the channel/dielectric interface or *s*‐SWNT/PNDI‐2T heterojunctions. With regard to the channel/dielectric interface, Zhang et al. reported that the localized states induced by gate bias possessed drawbacks leading to the accumulation of numerous trapped charges at the interface between channel materials and the dielectrics. This results in a substantial shift in the *V*_th_, even in an inert environment. To address this stability concern, the adoption of low‐*k* (low dielectric constant) nonpolar polymer dielectrics, including low‐*k* hydrophobic dielectrics such as SBS rubber, was proposed. The introduction of low‐*k* polymers aims to minimize the impact of gate polarization‐induced localized states, thereby reducing the quantity of fixed charges at the channel/dielectric interface. The low‐*k* property of these materials implies a decreased susceptibility to polarization under an electric field, contributing to the mitigation of fixed charge formation. The mentioned hydrophobic dielectric, SBS, is highlighted for its ability to eliminate the influence of moisture, oxygen, mobile charges, and impurities, allowing a lower bias instability or hysteresis‐free operation at the channel/dielectric interface.^[^ ^38^ ^]^

To confirm that the memory effect occurs at the interface between PNDI‐2T and *s*‐SWNTs rather than the electron defects within PNDI‐2T, a reference device comprising pure PNDI‐2T was fabricated and characterized. The transfer characteristics in the electrical writing state and OFF state of pristine PNDI‐2T are illustrated in Figure S17 (Supporting Information). As can be seen, there is no significant change in *V*_th_ after the electrical writing. The outcome demonstrates that PNDI‐2T exhibits electron‐defect‐free behavior. Therefore, the memory behavior should occur at the interface between *s*‐SWNT/PNDI‐2T. The unique memory effects are attributed to the supramolecular channel with plenty of charge‐transfer complex between *s*‐SWNT and PNDI‐2T. In Figure 3c, the output curve is conducted under the initial/photoerasing and electric writing states. It is obvious that the drain current can be effectively promoted after gate bias was previously applied. The previous results indicate that the structure of *s*‐SWNT/PNDI‐2T performs an excellent charge‐storage ability. To give a more comprehensive discussion about charge trapping behavior, we introduced different electrical writing *V*_g_s from 10 to 100 V. In Figure 3d, the transfer curves shifted to the positive side as the positive electrical writing voltage was applied. It is noteworthy that employing various programming regimes, involving changes in electrical writing *V*_g_, enables the realization of multiple discrete electrical states within a single device. The capability to achieve such diversity is a crucial prerequisite for designing multibit memory elements intended for high‐density information storage. This flexibility not only allows memory devices to switch between different states but also provides greater flexibility for efficient information storage.^[^ ^39^, ^40^, ^41^ ^]^

**Figure 4:**
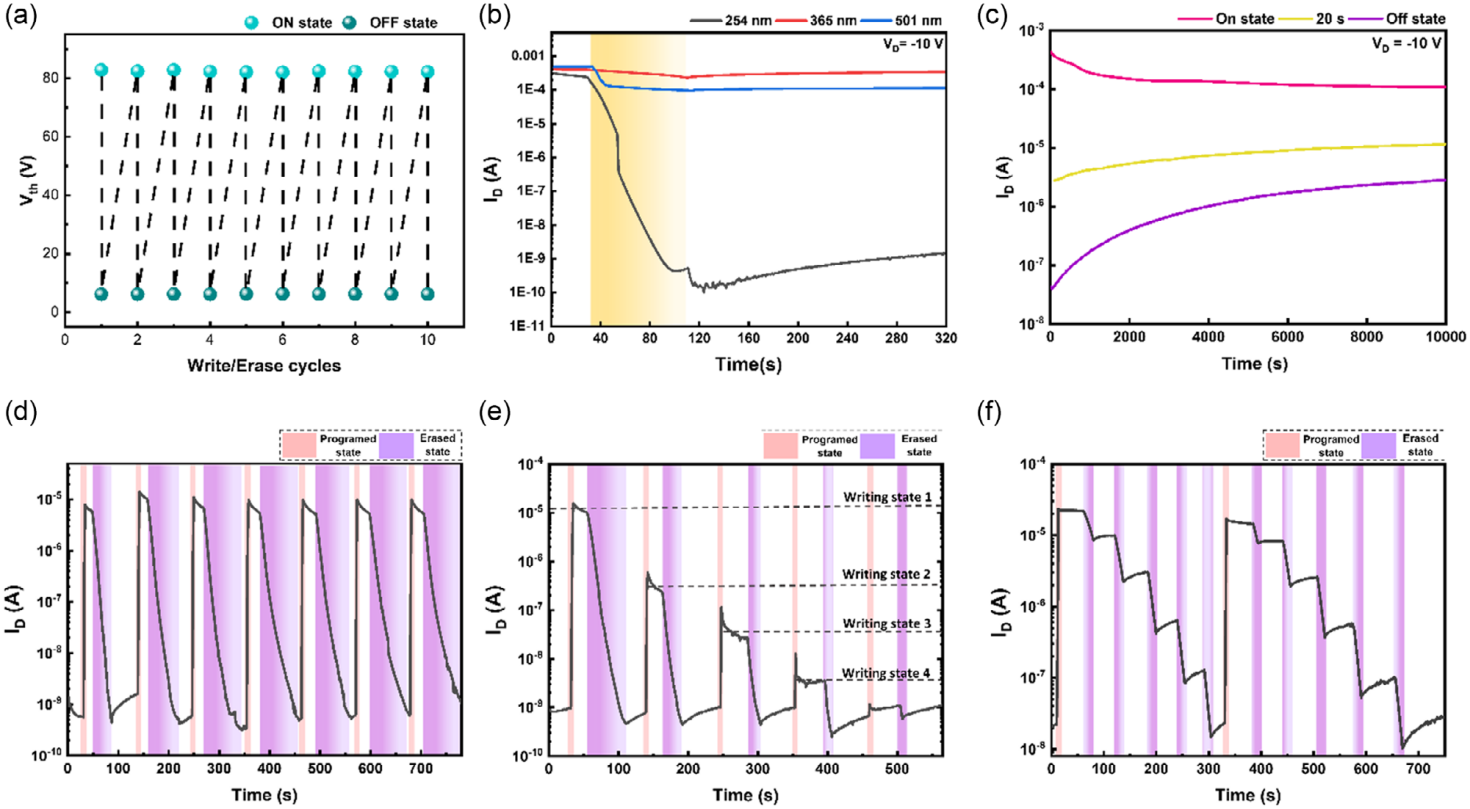
a) Threshold voltage variations of the phototransistor memory after consecutive electrical writing (*V*_g_ = 100 V; 1 s) and photoerasing (254 nm; 0.1 mW cm^−2^) cycles. b) Transient photocurrent characteristics of the memory device with different wavelengths of photoerasing (254 nm, 0.1 mW cm^−2^; 365 nm, 0.1 mW cm^−2^; 501 nm, 1 mW cm^−2^) for 80 s. c) Long‐term retention test of the memory device with electrical writing (ON state; *V*_g_ = 100 V; 1 s) and photoerasing (OFF state; 254 nm; 0.1 mW cm^−2^; 20 or 80 s). d) WRER measurements of the memory devices with electrical writing (ON state; *V*_g_ = 30 V; 1 s) and photoerasing (OFF state; 254 nm; 0.1 mW cm^−2^; 60 s). Multilevel memory behaviors driven by varied e) electrical erasing bias of 60, 30, 20, 10, and 5 V for 1 s, and f) accumulated photoerasing periods spanning the range of 10–50 s (254 nm, 0.1 mW cm^−2^). Note that all photocurrent measurements were conducted at *V*_d_ = −10 V.

To further investigate the photoresponse of the memory device, UV light (254 nm) and red light (617 nm) are applied. The results are shown in Figure 3e and S18 (Supporting Information). The photoerasing process was applied at *V*_d _= –10 V. As shown in the figures, the photoerasing process was conducted under different periods of time after the electric writing was performed by applying *V*_g_ of 100 V for 1 s. It can be seen that UV light exhibited better photoerasing ability. The device presented a stronger photoresponse within the 254 nm light illumination than the 617 nm. Under 80 s photoerasing, UV light could demonstrate the large Δ*V*_th_ = 75.7 V, bringing the transfer curve to return to the initial state (Table S2, Supporting Information). However, the Δ*V*_th_ of red light after 80 s illumination could only display the value of 29.4 V. Furthermore, the current modulation of UV‐light erasing can reach as high as electrical erasing with comparably high *I*_ON_/*I*_OFF_ of 10^5^. Furthermore, **Figure** **4**a and S19 (Supporting Information) depict the device correlation of the threshold voltage after consecutive write/erase cycles corresponding to the ON/OFF state. As can be seen, the memory device presented a stable memory switching and Δ*V*_th_ with high contrast between the ON and OFF states.

Multilevel memory behavior is a representative feature of phototransistor memory. Therefore, a series of control parameters were applied in terms of illumination time and light wavelengths to investigate the optoelectronic properties and device performance further. To further determine the obtainable memory states of the photomemory, transient characteristics of the device were evaluated at *V*_d _= –10 V and applied with 10, 20, 40, and 80 s photoerasing. As can be seen in Figure 3d, the *I*_ON_/*I*_OFF_ is ≈10, 10^3^, 10^4^, and 10^5^, respectively. The interesting aspect of this phenomenon lies in the diverse levels of OFF‐state current, which are conferred by different times of light illuminations, and it serves as a pivotal advantage for the practical implementation of multibit data storage applications. Moreover, it enhances the data discriminability of phototransistor memory systems, showcasing its potential to provide a highly nuanced and versatile platform for data storage and retrieval. As shown in Figure 4b, weak photoerasing effects were observed by applying 365 and 501 nm lights. However, at 254 nm, UV light exhibited the most pronounced erasing effect, achieving orders of magnitude close to 10^6^ after 80 s illumination. The charge modulation induced by the positive gate bias in the device was erased entirely after 80 s of illumination.

### 2.6 Long‐Term Stability and Switching Endurance of the Phototransistor Memory

The memory device's long‐term retention test was conducted at –10 V for 10^4^ s. As can be seen in Figure 4c, the ON state was achieved by applying *V*_g_ of 100 V for 1 s, and the OFF states were obtained by using 254 nm light illumination for 20 or 80 s. At the end of the test, the resulting memory device exhibited a stable *I*_ON_/*I*_OFF_ ratio of 10^3^, warranting its good performance in terms of long‐term stability. However, the *I*_ON_/*I*_OFF_ retentions were not stable and the ratios decayed from ≈10^6^ to ≈10^3^ within the first 1000 s. This unfavorable outcome is mainly caused by the lack of an electret layer within the photomemory structure. In photomemory devices containing an electret layer, electrons are stored at the interface between the electret layer and the semiconductor channel layer, and this arrangement is more stable than the design in this study.^[^ ^16^ ^]^ Therefore, the stored electrons may potentially recombine with hole carriers in the channel, leading to unstable *I*_ON_/*I*_OFF_ retentions. To further gain insight into the interaction between the stored electrons and hole carriers, the retention test was prolonged to 100 000 s in Figure S20 (Supporting Information). Though the current in the photoerased (OFF) states slightly increased in comparison to that in the 10 000 s retention test, the resulting memory behavior demonstrated a stable *I*_ON_/*I*_OFF_ ratio > 10^2^, presenting its long‐term stability. Next, UV light was employed for the erasing process due to its superior efficiency. Hence, the transistor memory device was tested by the write–read–erase–read (WRER) operation cycle to study its reproducible and reversible stability. As shown in Figure 4d, the WRER cycles were conducted at *V*_d_ = –10 V. First, the device was programmed under a *V*_g_ of 30 V for 1 s. Subsequently, the device was read at *V*_d_ = –10 V without applying gate bias. Finally, the erasing process was conducted by applying light illumination for 60 s, which removed the stored charges within the interface between CPs and *s*‐SWNTs. As can be seen in the figure, the *I*_ON_/*I*_OFF_ could achieve a constant level of 10^5^ under the rewriting procedure, and this device showed decent repeatability along the WRER cycles. In addition, the different stages of electric writing were applied with different *V*_g_ in the range of 5–60 V, as shown in Figure 4e. The device demonstrated excellent stability and multilevel ON states, as the drain current can switch to different levels by applying the corresponding magnitude of gate bias. Moreover, in Figure 4f, the memory device was programmed with a constant *V*_g_ of 60 V and erased by different UV light times to obtain different stages. This device phenomenon exhibited multilevel OFF states, which were triggered by varying durations of light exposure. Therefore, this evidences the multilevel behavior of the photomemory devices employing *s*‐SWNT/PNDI‐2T supramolecules. This characteristic provides a vast advantage for the practical use of multibit data storage applications. Furthermore, it significantly enhances the ability to differentiate data in phototransistor memory systems. This not only demonstrates its potential to offer an extremely sophisticated and flexible platform for storing and retrieving information but also enables the reading of accessed data without the need to apply *V*_g_, achieving a nondestructive readout and providing superior stability.

### 2.7 Working Mechanism of the Memory Device

**Figure 5:**
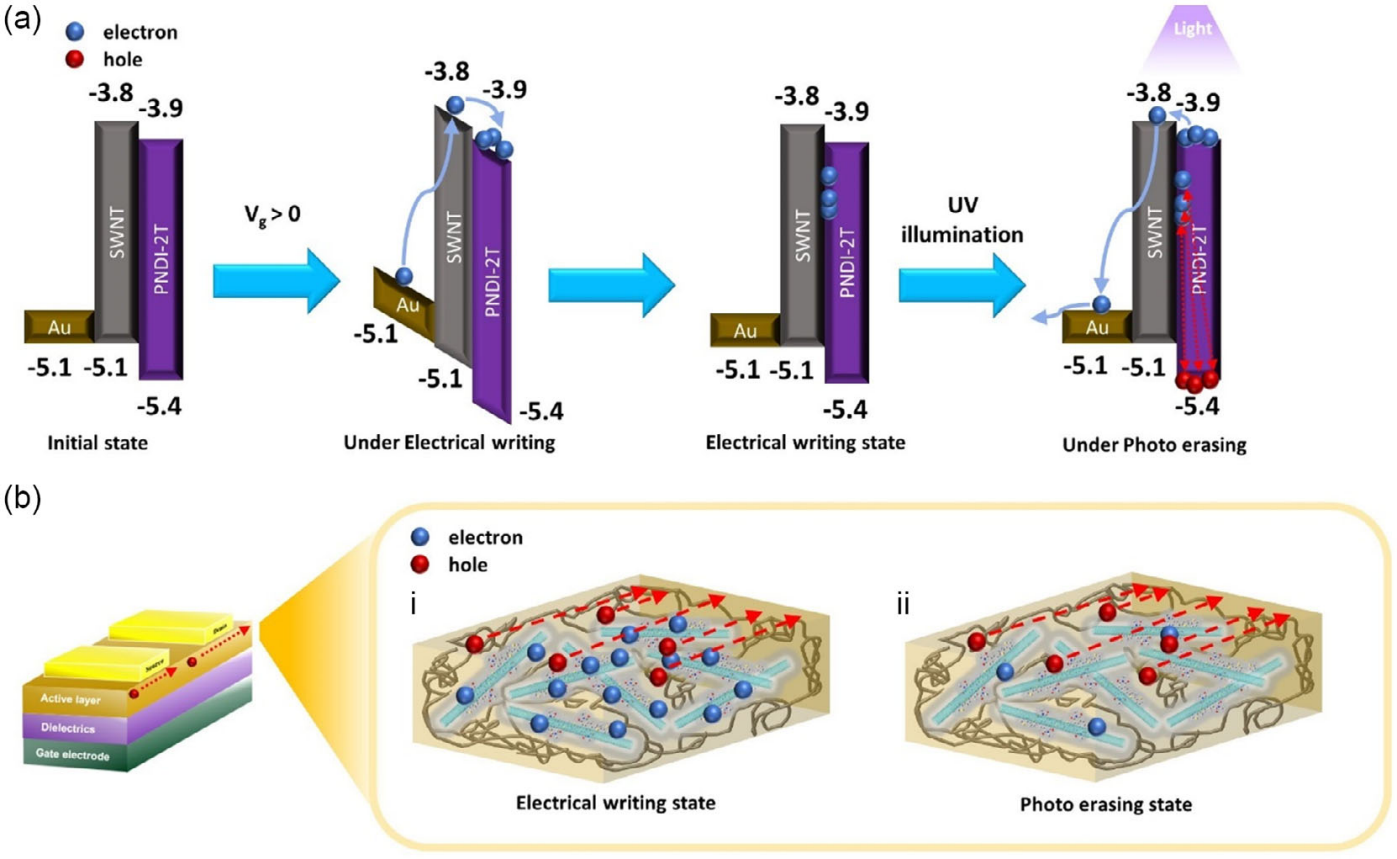
a) Schematic working mechanism and energy level alignments of the constituent materials in the phototransistor memory. b) Charge trapping illustration of the *s*‐SWNT/CP supramolecules in phototransistor memory device: i) during electrical writing, the electrons were trapped in the supramolecules and at the heterojunction interfaces of *s*‐SWNT/CP, and the counter holes were transported through the *s*‐SWNT channel to switch the memory into ON state; ii) during photoerasing, the trapped electrons were excited and removed from the *s*‐SWNT/CP interfaces to switch the memory into OFF state.

To gain insight into the “electrical‐ON” and “photo‐OFF” memory behavior in the *s*‐SWNT‐based FET device, a plausible working mechanism based on the *s*‐SWNT/CP supramolecules is proposed. **Figure** **5**a demonstrates the energy level alignments of the constituent materials in the phototransistor memory. The energy levels of the constituent materials are referred to in the literature.^[^ ^42^, ^43^, ^44^ ^]^ During the electrical writing process with positive *V*_g_ applied, as shown in the figure, electrons are injected into the active layer. With more and more electrons filled in the active layer, they are trapped by the charge‐transfer complex in the supramolecule channel, thereby inducing a positively shifted threshold voltage. Hence, electrons are stored within the interface between the CPs and the SWNTs, demonstrating the electrical writing state. Under the photoerasing process with UV‐light illumination, CPs can absorb specific wavelengths of light, such as 254 and 617 nm, depending on their optical absorption. Subsequently, excitons can be generated by absorbing the energy of light. After exciton dissociation, holes are recombined with the previously injected/trapped electrons within the interface between *s*‐SWNT/PNDI‐2T and recover the charge modulations, thereby inducing a negatively shifted threshold voltage, and the device is recovered into the photoerased (OFF) state. This exciton‐driven mechanism is described in Figure 5a. However, the neutrality in the channel is necessary to be balanced by the applied drain voltage. Another potential mechanism is that the trapped electrons can be excited by the photon energy. When the absorbed energy surpasses the energy barrier of electron‐trapping defects, electrons can be released from the interface between *s*‐SWNT/PNDI‐2T. This charge‐releasing process leads to the restoration of charge modulations and the device can be switched back to the OFF state. However, the existence of the trapped electron excitation may need further investigation.

The structure–performance relationship of the supramolecule channel is next discussed. As shown in Figure 5b, electrons are trapped within the s‐SWNT/PNDI‐2T interface in the electrical writing state while holes are transported between the source/drain channel electrodes. Under light illumination, the trapped electrons are removed from the heterojunction interface. It is worth noting that the charge‐transfer complex between *s*‐SWNT and PNDI‐2T in the supramolecules didn't disrupt the charge transport in the channel; instead, they endowed the device with intrinsic charge‐trapping sites at the heterojunction interfaces and light‐driven gating capability. Therefore, PNDI‐2T, with an optimized donor spacer, coplanarity, and aggregating ability, forms the mostly stable supramolecular network with *s*‐SWNT to achieve the highest yield among the n‐type CPs in this study.

## 3 Conclusion

In conclusion, NDI‐based polymers were studied and applied to wrap SWNTs selectively. Based on the optical characterizations and molecular simulations, CPs with a larger donor unit indicate poor chain coplanarity and potentially better chain aggregating capability due to the flexible conjugated chains. However, PNDI‐2TF shows much lower sorting efficiency than PNDI‐2T, possibly because of the additional steric hindrance from the fluorine atom on the donor unit, thereby hindering the wrapping on *s*‐SWNTs. Eventually, PNDI‐2T has adequate steric hindrance and poor coplanarity to make the chains with sufficient surface available to contain the SWNTs. With favorable spatial allocation and order graphitic structure, the enriched *s*‐SWNT content and heterojunction interfaces between *s*‐SWNT and PNDI‐2T can conduce to improving the charge‐trapping ability and photoresponse in phototransistor memory. It is worth mentioning that the charge‐transfer complex between *s*‐SWNT and PNDI‐2T in the supramolecules didn't disrupt the charge transport in the channel. The device still achieves a high *μ*_h_ of 0.50 and 2.53 cm^2^ V^−1^ s^−1^ at *V*_d_ = –10 and –100 V. In contrast, the unique memory effects are attributed to the supramolecular channel with plenty of charge‐transfer complex between *s*‐SWNT and PNDI‐2T. Therefore, the device exhibited a high *I*_ON_/*I*_OFF_ and Δ*V*_th_ of 10^5^ and 75.7 V, representing its remarkable charge‐storage capabilities. In addition, the device demonstrates decent long‐term stability over 10^4^ s and multilevel memory behavior driven by the varied gate bias or accumulated light‐gating periods. The proposed memory device combines electrical writing/erasing and photoerasing processes along with nondestructive readout and potential multibit data storage, offering insights into the development of *s*‐SWNT‐based FET memory. In summary, this research contributes to developing advanced phototransistor memory devices, paving the way for future electronic applications.

## 4 Experimental Section

4.1

4.1.1

##### Materials

NDI‐based polymers with different donor units of thiophene (T), thienothiophene (TT), selenophene (Se), bifluorothiophene (2TF), and bithiophene (2T) were synthesized using the reported method and described in Supporting Information (Scheme S1, Supporting Information).^[^ ^45^ ^]^ Note that the donor monomers were purchased from Luminescence Technology Corp. Dextran, PMMA, phenylbis(2,4,6‐trimethylbenzoyl) (97%), and pentaerythritol tetrakis(3‐mercaptopropionate) (>95%) were purchased from Sigma‐Aldrich. SBS, toluene, acetone, PD SWNTs, and 1‐CN were purchased from Yuang Hong Inc., J. T. Baker, DUKSAN, NanoIntegris, and TCI, respectively. All the purchased chemicals were used as received without further purification.

##### Selective Sorting Procedure of s‐SWNTs

The n‐type CP of 5 mg was fully dissolved in toluene (20 mL) using ultracleaner EASY 60H (Elma, Inc.) for 5 min. Next, PD SWNTs (20 mg) were added with a weight ratio of CP/SWNTs = 1:4. Note that the CPs served as dispersants of SWNTs. The mixtures were sonicated with 40% amplitude for 30 min using a Q700 (QSONICA, Inc.), and isopropanol was used to maintain an average temperature of –60 °C. Next, the sorting solutions were ultracentrifuged at 17 000 rpm and 20 °C for 1 h. Finally, the liquor supernatant with enriched *s*‐SWNTs was separated from the precipitated solid.

##### Fabrication of Photomemory Transistors

To fabricate phototransistor memory with a BG/TC configuration, we first prepared a solution with 300 mg of dextran dissolved in 10 mL water (30 mg mL^−1^), and then the solution was spin‐coated onto a bare wafer at 1500 rpm for 1 min. After the spin‐coating process, the wafer was heated at 140 °C for 10 min to evaporate the residual water. Next, the solution comprising *s*‐SWNT sorted by PNDI‐2T was diluted with the ratio of sorting solution/fresh toluene = 2:1 (vol:vol), and the dextran‐coated wafer was soaked in the diluted solution for 3 days. After the *s*‐SWNT adsorption process, the wafer was washed with toluene and soaked in toluene for 10 min to remove excess polymers. The wafer was then spin‐coated (1500 rpm, 1 min) with PMMA (40 mg mL^−1^ in toluene) to conduct the film‐transfer process. Note that PMMA was an agent to adsorb the *s*‐SWNT/PNDI‐2T macromolecular film. The wafer was cut into pieces, and the bilayered PMMA/*s*‐SWNT film was fixed with commercial tapes and peeled off by immersing the wafer in water. Next, SBS, phenyl bis(2,4,6‐trimethylbenzoyl), and pentaerythritol tetrakis(3‐mercaptopropionate) with a weight ratio of 25:1:1 were dissolved in toluene (solid content: 1.6 wt%). The solution was spin‐coated onto another wafer with a 300 nm thick SiO_2_ layer, and the film was cured to crosslink the SBS and serve as a polymer dielectric. Subsequently, the bilayered PMMA/*s*‐SWNT film was transferred onto the SBS layer. The wafer was washed with acetone to remove the PMMA film. Finally, a 30 nm thick Au layer was thermally deposited through a patterned shadow mask with channel length (*L*) and width (*W*) of 100 and 2000 μm, respectively, to define the top‐contact electrode.

##### Characterization

The absorbance of the solutions containing the CPs/PD SWNTs hybrid and the aggregation of CPs were examined using UV–Vis–NIR spectroscopy, employing U‐4100 (Hitachi), scanning in the range from 400 to 1600 nm. To identify the degree of aggregation of CPs in toluene, the polymer was dissolved in 1‐CN and toluene at 0.05 and 0.25 mg mL^−1^. Note that the CP in 1‐CN was defined as the disorder fraction, and the aggregation fraction of CP in toluene could be obtained by comparing the optical absorption of two samples. The calculation of aggregation fraction was adapted from a reported procedure.^[^ ^46^, ^47^ ^]^ The Raman spectrum of the *s*‐SWNT/CP film on a glass substrate was obtained using a Gloucestershire GL12 8JR (RENISHAW) with an excitation wavelength of 633 nm. The FTIR spectrum of the *s*‐SWNT/CP film on a glass substrate was obtained using a Nicolet 6700 (Thermo Scientific), scanning from 1000 to 4000 nm. The surface topologies of the *s*‐SWNT/CP thin film were examined using a Dimension Icon AFM (Bruker) under the tapping mode at room temperature. All electrical characterizations were performed using a Keithley 4200‐SCS semiconductor parameter analyzer. The testing environment was maintained in darkness and ambient conditions. The areal capacitance (*C*_total_) of the SiO_2_ and SBS‐bilayered dielectrics^[^ ^48^ ^]^ was calculated using Equation (1), and *s*‐SWNT/PNDI‐2T supramolecules were measured at 2 kHz using a Keithley 4200‐SCS semiconductor parameter analyzer equipped with a digital capacitance–voltage measurement unit. Gold electrodes were directly deposited onto the electret layer following a metal–insulator–metal architecture to create the test structure. Hole mobility (*μ*_h_) and threshold voltage (*V*_th_) were calculated from the slope or extrapolation of the square root of source‐to‐drain current (*I*_ds_^1/2^) versus gate voltage (*V*_g_) in the saturation regime of the transfer curve, as shown in Equation (2).

(1) $$\frac{1}{C_{\text{total}}}=\frac{1}{C_{{\text{SiO}}_{2}}}+\frac{1}{C_{\text{polymer}}}$$

(2) $$I_{\text{ds}}=\frac{WC_{\text{total}}\mu_{h}}{2L}{\left(V_{g}-V_{\text{th}}\right)}^{2}$$

##### Molecular Simulation

DFT calculations were employed using Gaussian 09 W software with the Becke, 3 parameter, Lee‐Yang‐Parr (B3LYP) method and a 6–31G basis set to determine the optimized ground state configuration and molecular structure of the CPs studied. Note that 2 acceptors and 3 donors were connected to represent the polymer's repeating unit, and their side chains on the acceptor unit were replaced with a methyl group to simplify the optimization. MD calculations were applied to evaluate the interaction between the CP and SWNT. The calculations followed a reported procedure.^[^ ^49^, ^50^ ^]^ In this study, simulations focused on CPs interacting with armchair (9,9) semiconducting SWNTs in a vacuum. SWNT length was set as 19.6 nm (12 repeats). Molecular structures of CPs were generated by Avogadro and auto‐optimized using an Merck molecular force field 94 static vatiant (MMFF94s) force field.^[^ ^51^ ^]^ Under the software framework of Material Studio, the COMPASSIII force field was applied to all processes of CPs except PNDI‐Se using a universal force field.^[^ ^52^, ^53^ ^]^ Next, by using an adsorption locator tool to simulate the attachment of CP on the SWNT surface, the atom‐based van der Waals and the short‐range electrostatic interactions were evaluated with a cutoff of 2 nm. The long‐range electrostatic potential was computed by the group‐based Ewald summation method.^[^ ^54^ ^]^ The simulation was conducted by applying four annealing cycles with 15 000 steps per cycle.

## Conflict of Interest

The authors declare no conflict of interest.

## Supporting information

Supplementary Material

## Data Availability

The data that support the findings of this study are available from the corresponding author upon reasonable request.
