# Supplementary material for: Semiconducting Carbon Nanotubes with Light‐Driven Gating Behaviors in Phototransistor Memory Utilizing an N‐Type Conjugated Polymer Sorting
Source: Small Sci. 2024 Feb 22;4(4):2300268. doi: 10.1002/smsc.202300268 (PMC11935074; doi:10.1002/smsc.202300268)
Supplement: Supplementary file 1 — Supplementary Material [file SMSC-4-2300268-s001.pdf]

Supporting Information

Semiconducting Carbon Nanotubes with Light-Driven Gating Behaviors in Phototransistor Memory Utilizing An N-type Conjugated Polymer Sorting

Yi-Hsuan Tung,[a] *Shang-Wen Su,*[a] *En-Jia Su,*[b] *Guo-Hao Jiang,*[a] *Chun-Chi Chen,*[a] *Sheng-Sheng Yu,*[a] *Chi-Cheng Chiu,*[a] *Chien-Chung Shih,*[b*] *and Yan-Cheng Lin*[a,c*]

Y.-H. Tung, S.-W. Su, G.-H. Jiang, C.-C. Chen, Prof. S.-S. Yu, Prof. C.-C. Chiu, Prof. Y.-C. Lin
[a] Department of Chemical Engineering, National Cheng Kung University, Tainan 70101, Taiwan.

E.-J. Su, Prof. C.-C. Shih
[b] Department of Chemical Engineering and Materials Engineering, National Yunlin University of Science and Technology, Yunlin 64002, Taiwan.

Prof. Y.-C. Lin
[c] Advanced Research Center for Green Materials Science and Technology, National Taiwan University, Taipei 10617, Taiwan

* E-mail: shihcc@yuntech.edu.tw (C.-C. Shih); ycl@gs.ncku.edu.tw (Y.-C. Lin)

General Procedure of Polymerization

The polymerization of the studied polymers is described as follows. 4,9-Dibromo-2,7-bis(2-octyldecyl)benzo[lmn][3,8]-phenanthroline-1,3,6,8-tetraone (**NDI**) was copolymerized with equivalent 2,5-bis(trimethylstannyl)thiophene (**T**), 2,5-bis(trimethylstannyl)selenophene (**Se**), 2,5-bis(trimethylstannyl)thieno[3,2-b]thiophene (**TT**), 5,5'-bis(trimethylstannyl)-2,2'-bithiophene (**2T**), or (3,3'-difluoro-[2,2'-bithiophene]-5,5'-diyl)bis(trimethylstannane) (**2TF**) with the existence of $Pd_2(dba)_3$ (11 mol% with respect to **NDI**), and P(o-tolyl)$_3$ (100 mol% with respect to **NDI**) in chlorobenzene (CB) at a concentration of 0.04 M. The polymerization was conducted *via* the Stille coupling reaction (**Scheme S1**) at 130 °C for 48 hr. The reaction mixture was cooled to room temperature after end-encapsulation with 2-(tributylstannyl)thiophene, followed by 2-bromothiophene at 130 °C for 6 hr, respectively. The crude polymer solution was then precipitated in methanol and purified by Soxhlet extraction with methanol, acetone, hexane, and chloroform in sequence. Finally, the polymer product was obtained by precipitating the polymer solution in methanol and filtering the precipitates.

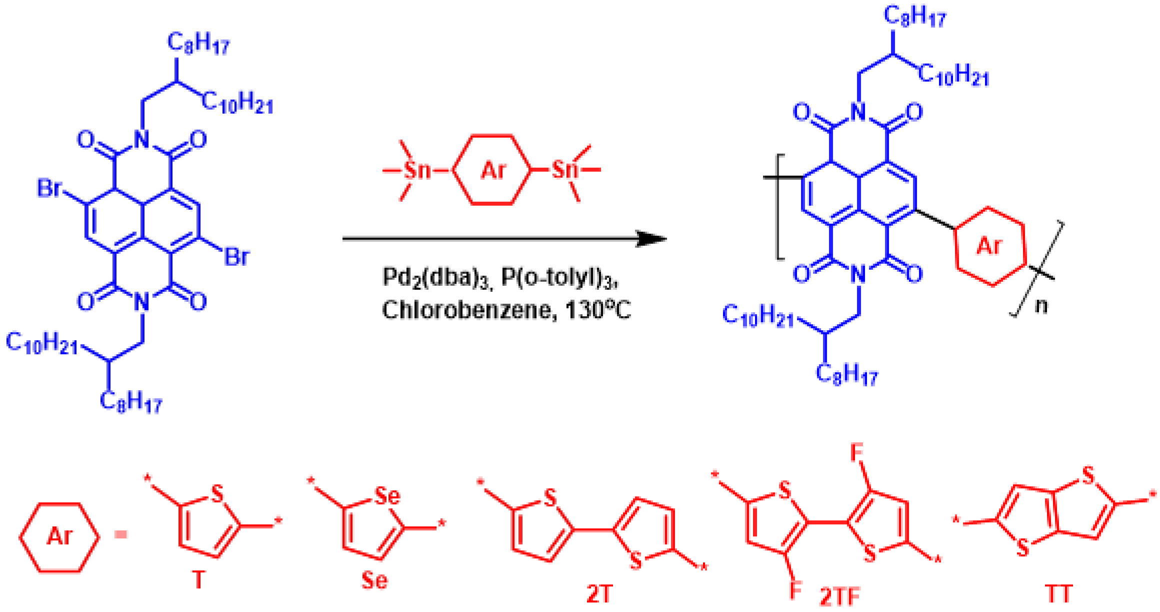

Scheme S1. Synthesis of the NDI-based CPs through Stille coupling reaction.

PNDI-T. NDI (230.0 mg, 0.21 mmol), **T** (86.0 mg, 0.21 mmol), $Pd_2(dba)_3$ (22.0 mg, 0.024 mmol), $P(o\text{-tolyl})_3$ (64.0 mg, 0.21 mmol), chlorobenzene (5 ml). Dark green solid (yield: 176 mg, 88%). Molecular weight evaluated by GPC (**Figure S1**; green line): M_n = 24,400, M_w = 60,600, PDI =2.49. ^{1}H-NMR in $CDCl_3$ (**Figure S2**).

PNDI-2T. NDI (705.0 mg, 0.64 mmol), **2T** (316.0 mg, 0.64 mmol), $Pd_2(dba)_3$ (58.8 mg, 0.064 mmol), $P(o\text{-tolyl})_3$ (195.0 mg, 0.64 mmol), chlorobenzene (16 ml). Blue solid (yield: 500 mg, 77.4%). Molecular weight evaluated by GPC (**Figure S1**; black line): M_n = 73,900, M_w = 146,000, PDI = 1.98. ^{1}H-NMR in $CDCl_3$ (**Figure S3**).

PNDI-TT. NDI (218.0 mg, 0.2 mmol), **TT** (101.0 mg, 0.22 mmol), $Pd_2(dba)_3$ (22.0 mg, 0.024 mmol), $P(o\text{-tolyl})_3$ (65.0 mg, 0.21 mmol), chlorobenzene (5 ml). Dark blue solid (yield: 145 mg, 73%). Molecular weight evaluated by GPC (**Figure S1**; purple line): M_n = 73,900, M_w = 135,600, PDI =1.83. ^{1}H-NMR in $CDCl_3$ (**Figure S4**).

PNDI-Se. NDI (230.0 mg, 0.21 mmol), **Se** (95.9 mg, 0.21 mmol), $Pd_2(dba)_3$ (22.0 mg, 0.024 mmol), $P(o\text{-tolyl})_3$ (64.0 mg, 0.21 mmol), chlorobenzene (5 ml). Dark green solid (yield: 176 mg, 88%). Molecular weight evaluated by GPC (**Figure S1**; orange line): M_n = 16,300, M_w = 40,700, PDI =2.50. ^{1}H-NMR in $CDCl_3$ (**Figure S5**).

PNDI-2TF. NDI (230.0 mg, 0.21 mmol), **2TF** (110.8 mg, 0.21 mmol), $Pd_2(dba)_3$ (22.0 mg, 0.024 mmol), $P(o\text{-tolyl})_3$ (64.0 mg, 0.21 mmol), chlorobenzene (5 ml). Dark green solid (yield: 176 mg, 88%). Molecular weight evaluated by GPC (**Figure S1**; red line): M_n = 90,600, M_w = 234,200, PDI =2.59. ^{1}H-NMR in $CDCl_3$ (**Figure S6**).

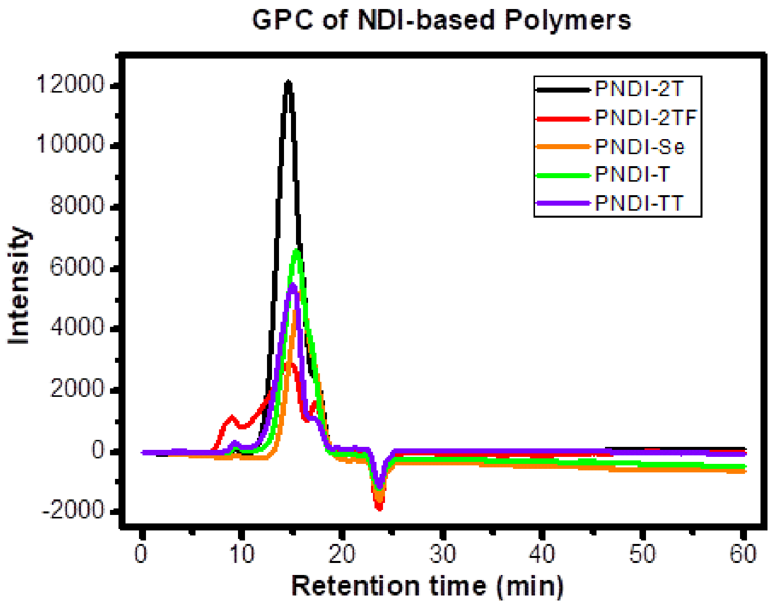

Figure S1. GPC profiles of the n-type CPs in THF at 40 °C and 1 mL min^{-1}. Note that the molecular weight measurements were referred to as standard polystyrene.

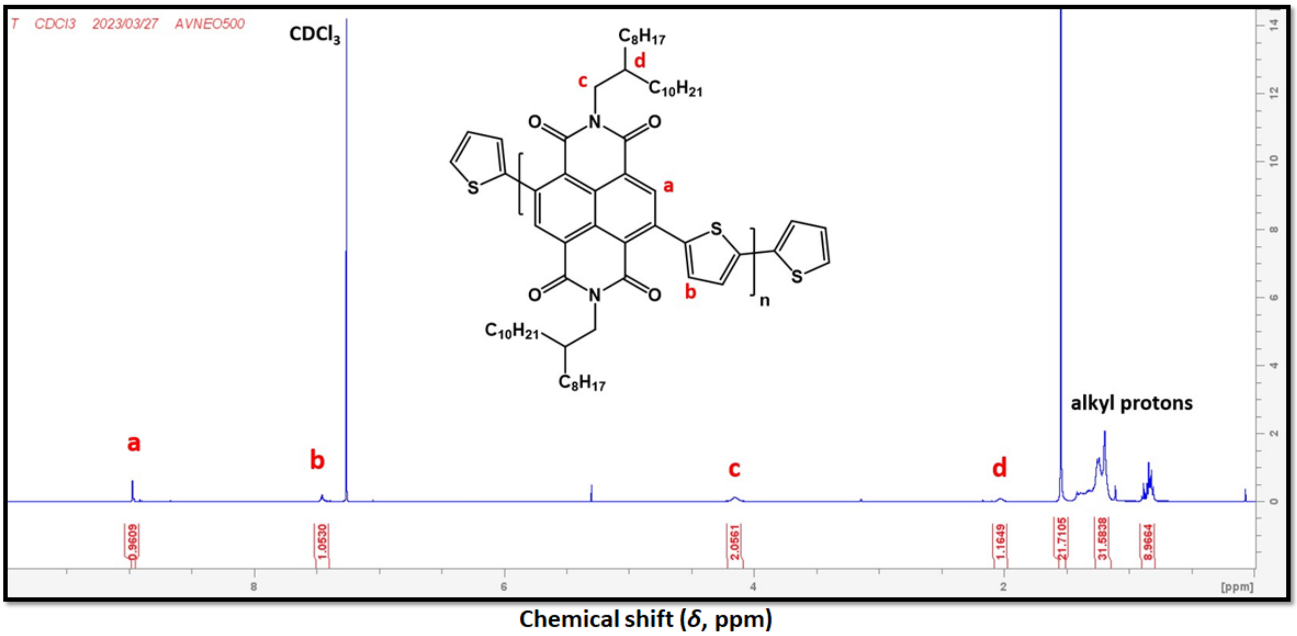

Figure S2. ^{1}H-NMR of PNDI-T in $CDCl_3$.

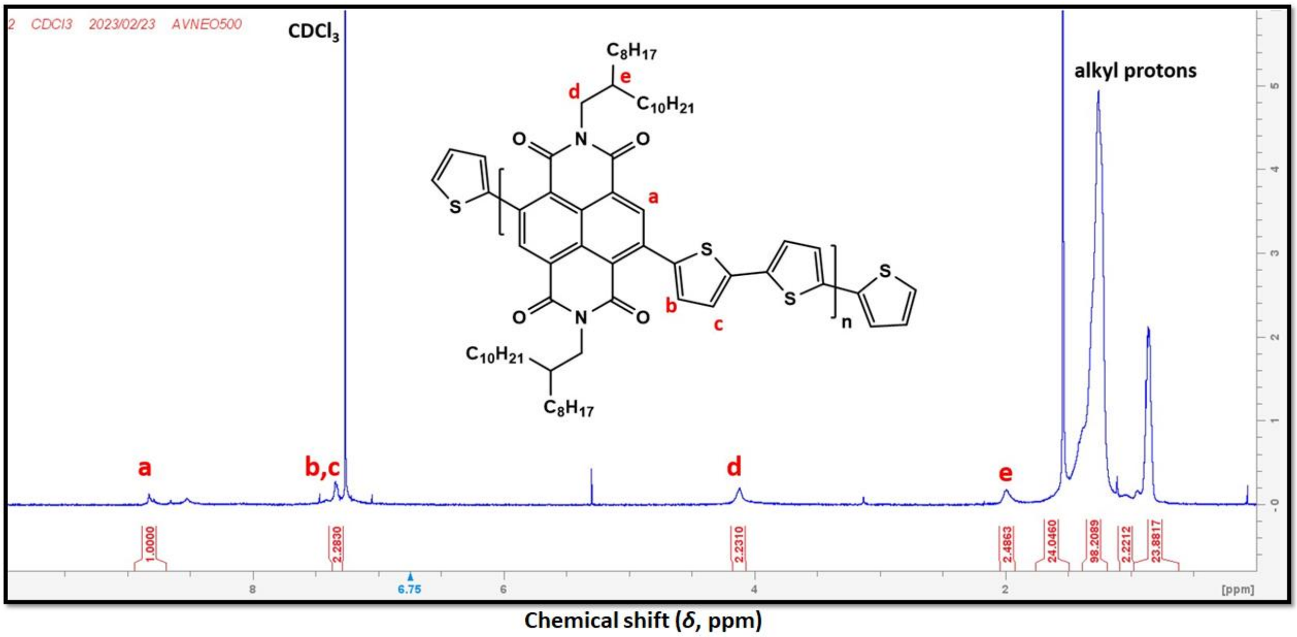

Figure S3. ^{1}H-NMR of PNDI-2T in $CDCl_3$.

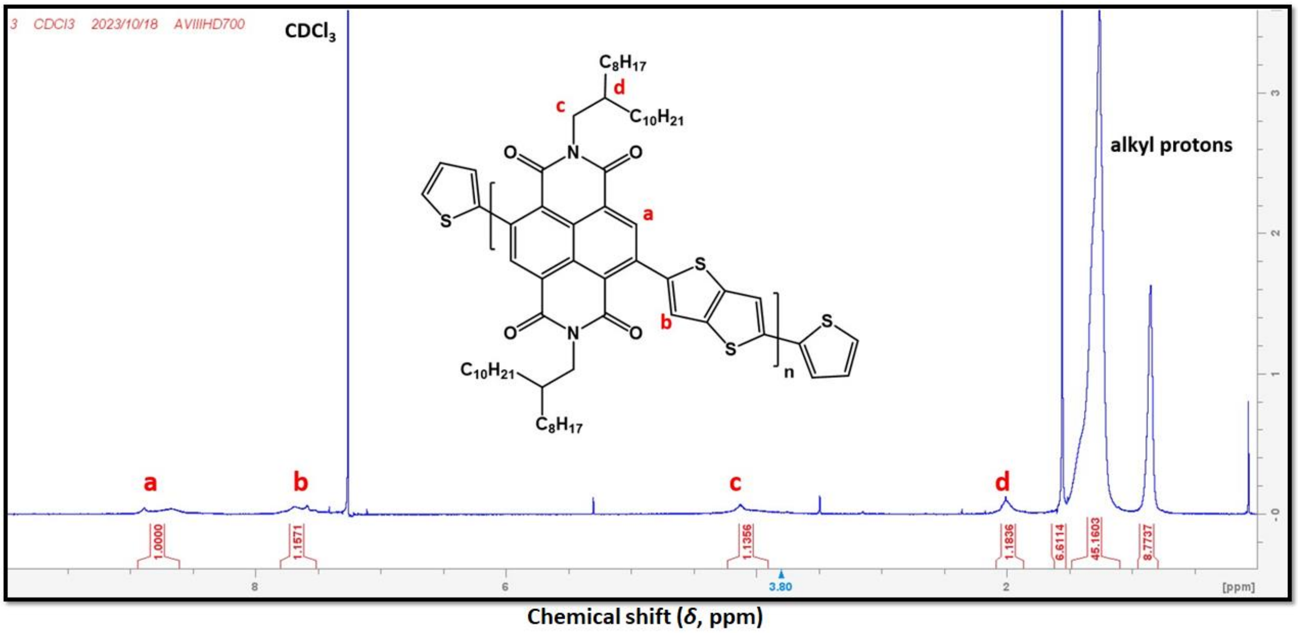

Figure S4. ^{1}H-NMR of PNDI-TT in $CDCl_3$.

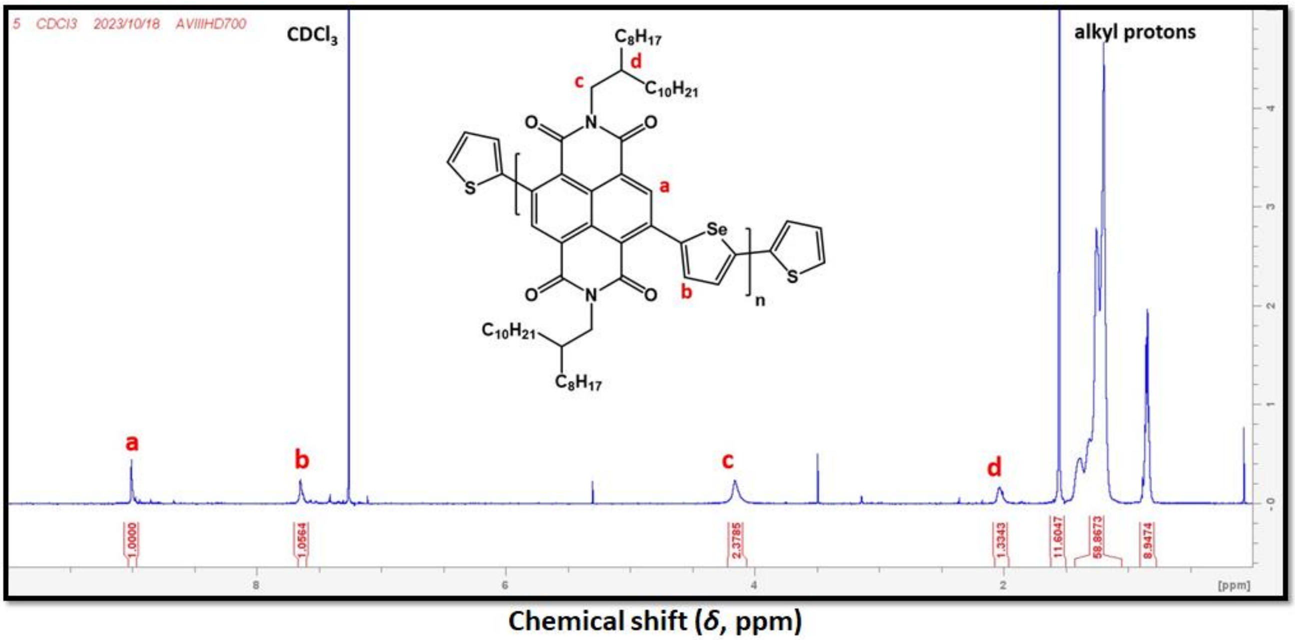

Figure S5. ^{1}H-NMR of PNDI-Se in $CDCl_3$.

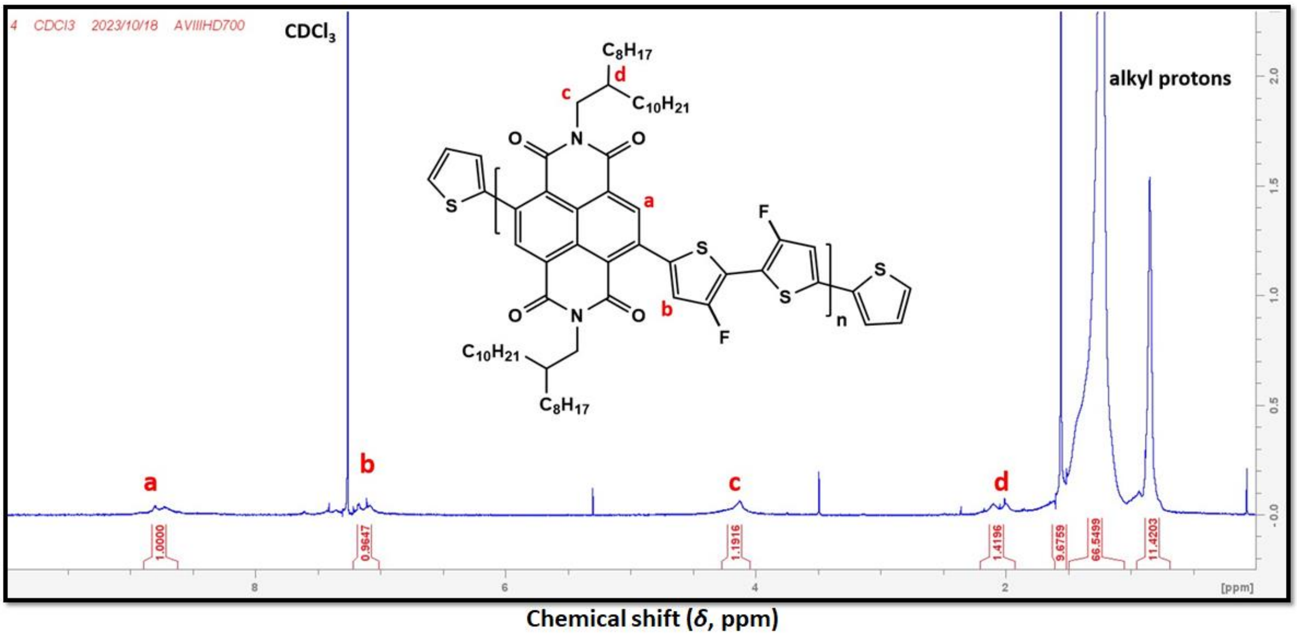

Figure S6. ^{1}H-NMR of PNDI-2TF in $CDCl_3$.

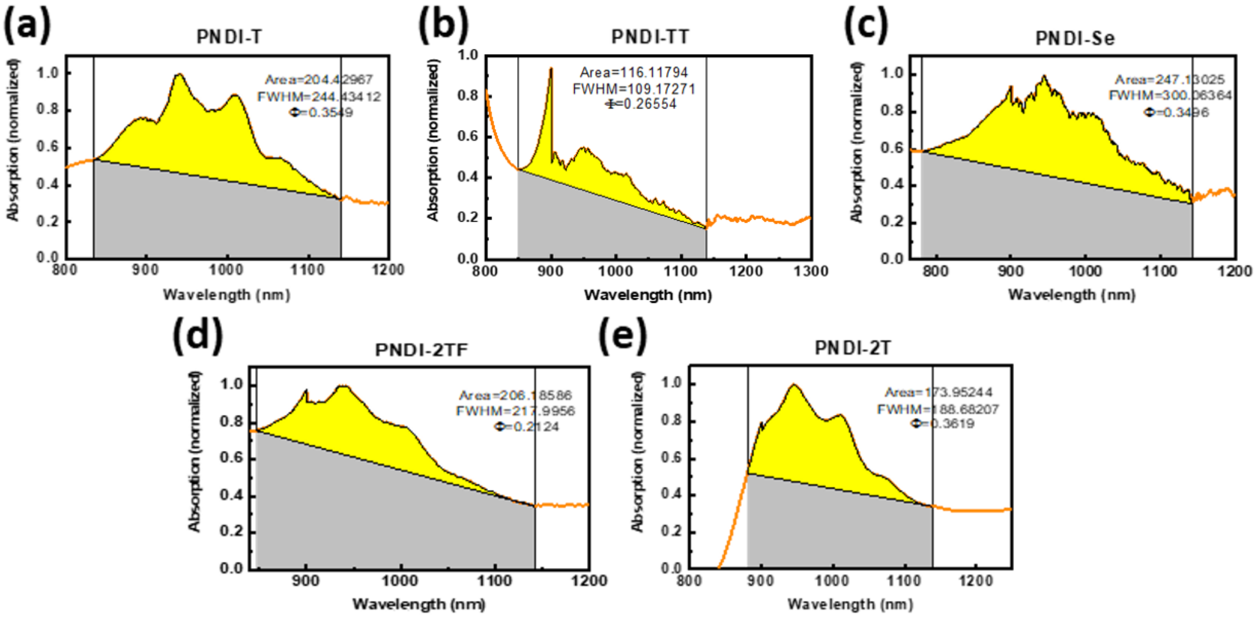

Figure S7. UV-vis absorption spectra of the sorted *s*-SWNT solutions with (a) PNDI-T, (b) PNDI-TT, (c) PNDI-Se, (d) PNDI-2TF, and (e) PNDI-2T in toluene to calculate the sorting selectivity.

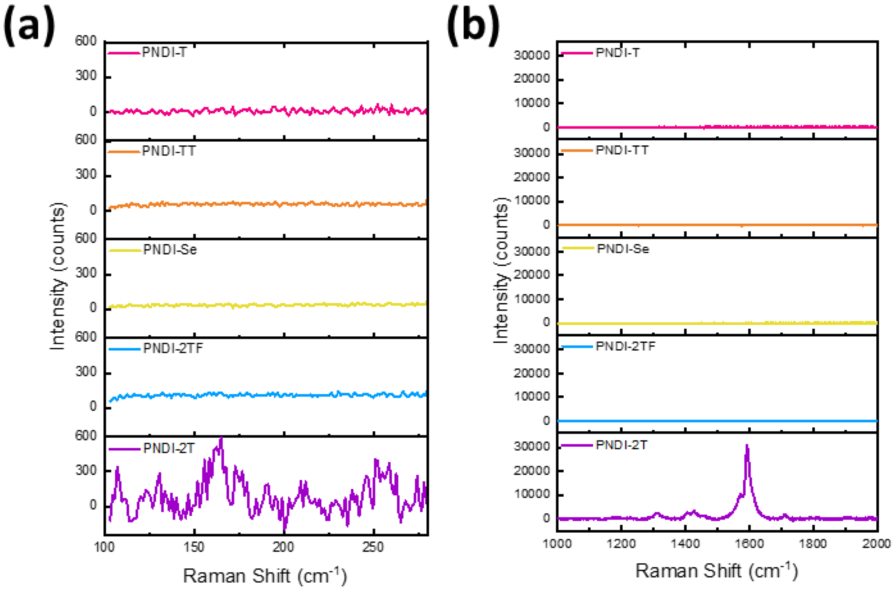

Figure S8. Raman spectra of pristine s-SWNT and *s*-SWNT sorted by n-type CPs with an excitation wavelength of 633 nm at different Raman shift bands spanning the range of (d) 100-300 nm or (e) 1000-2000 nm.

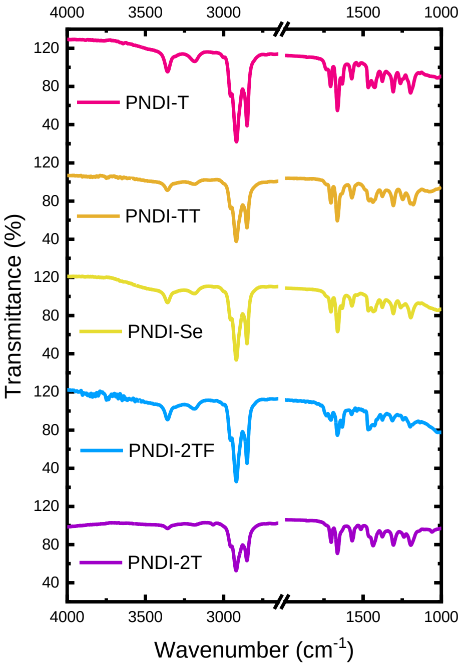

Figure S9. FT-IR spectra of the *s*-SWNT/n-type CP films.

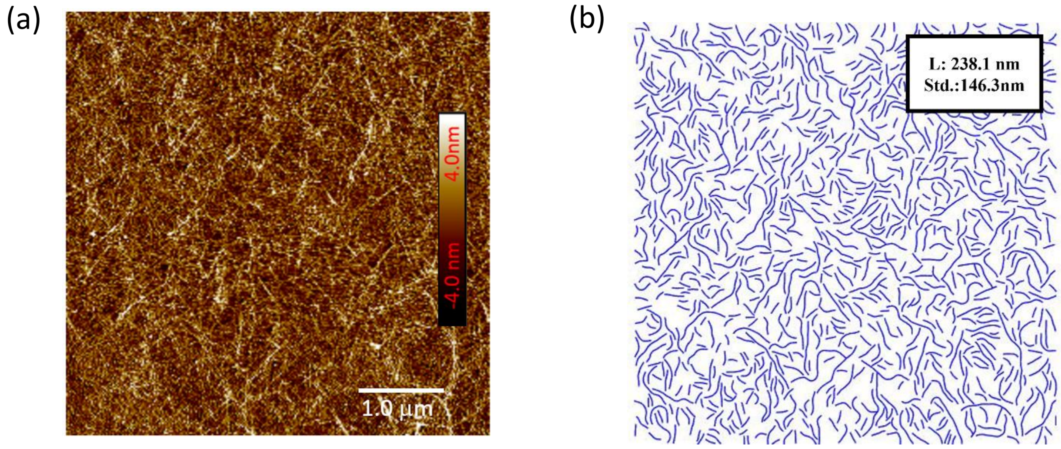

Figure S10. (a) AFM topography of the *s*-SWNT/PNDI-2T film derived from a diluted *s*-SWNT/PNDI-2T solution with a one-fourth concentration in the original process. (b) Fiber mapping of the AFM image by using the GTFiber software. The inset shows the average length and its standard deviation of the *s*-SWNTs.

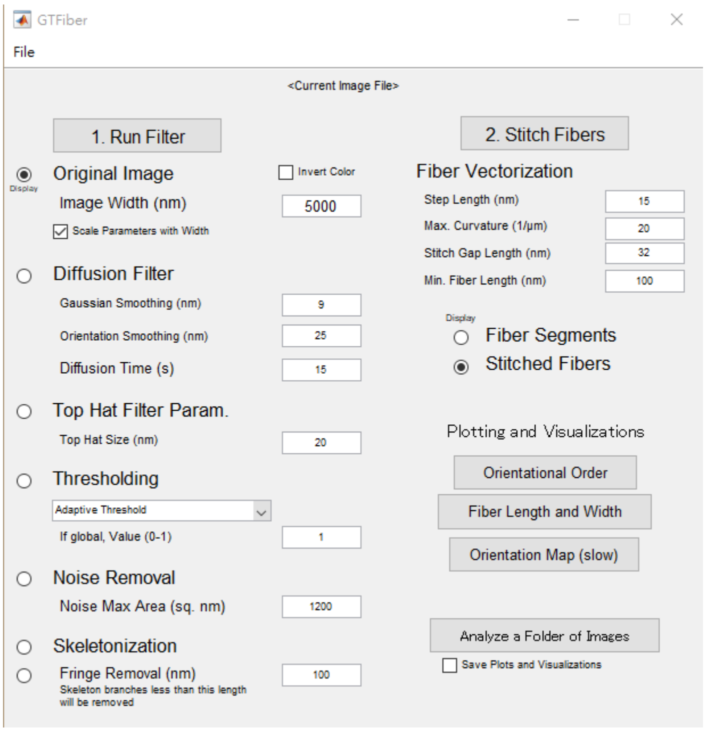

Figure S11. GTfiber parameters for the analysis of the *s*-SWNT's AFM topography used in this study. The software was developed by Persson, N. E.; McBride, M. A.; Grover, M. A.; Reichmanis, E. Automated Analysis of Orientational Order in Images of Fibrillar Materials. *Chem. Mater.* **2017**, *29*, 3−14.

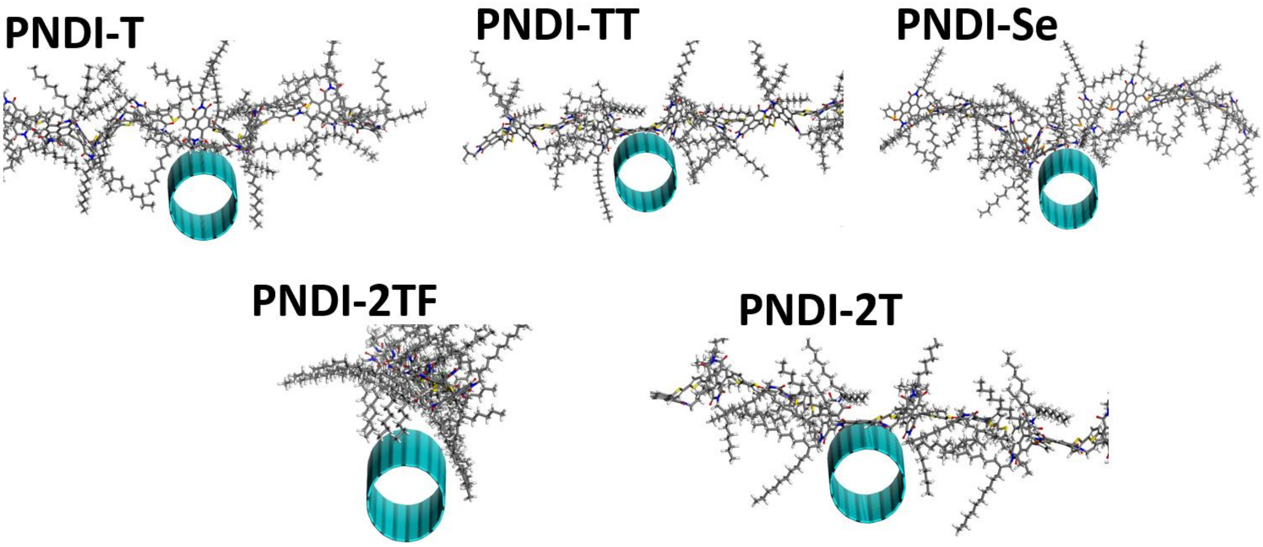

Figure S12. MD simulation snapshots in the top view to illustrate the π-π interaction between SWNTs and n-type CPs.

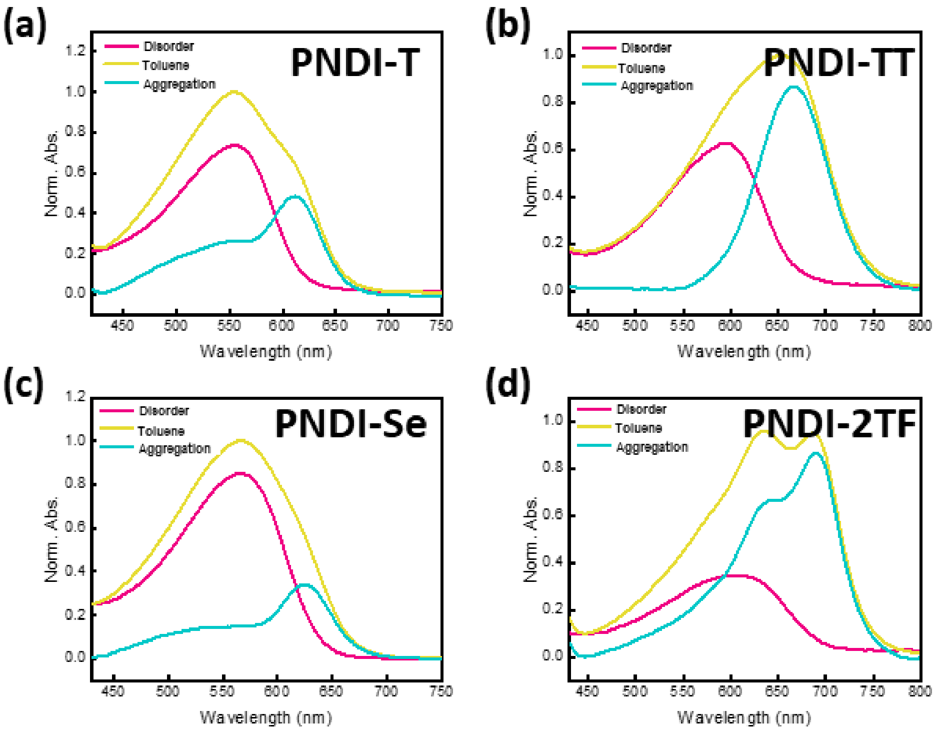

Figure S13. Deconvolution of the aggregation and disorder fractions in the UV−vis absorption spectra of n-type CP solutions. Note that the disordered state is defined from the polymer solutions of 1-CN at 0.05 mg mL^{-1}; the polymer solutions in toluene were prepared at a fixed concentration of 0.25 mg mL^{-1}.

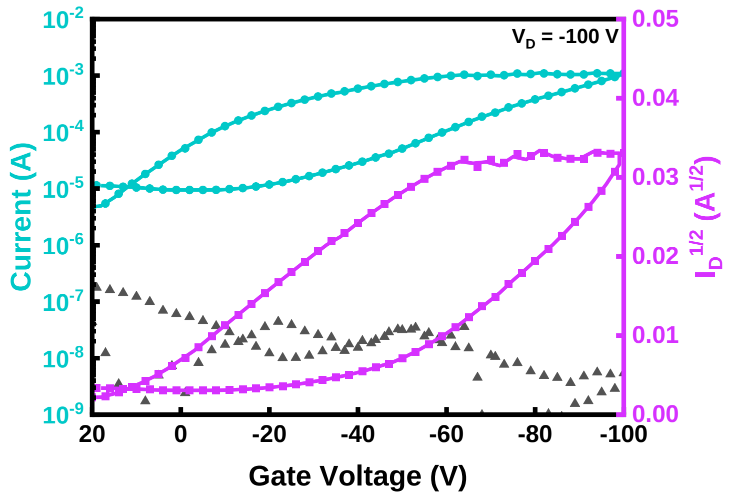

Figure S14. Transfer characteristics of the device comprising *s*-SWNT/PNDI-2T with V_D = –100 V and V_G swept from 20 to –100 V. Note that the cyan blue, gray, and purple lines indicate the drain current (I_D), gate current (I_G), and the square root of drain current ($I_D^{1/2}$). The calculated hole mobility (μ_h) is 2.53 cm^2 V^{-1} s^{-1}.

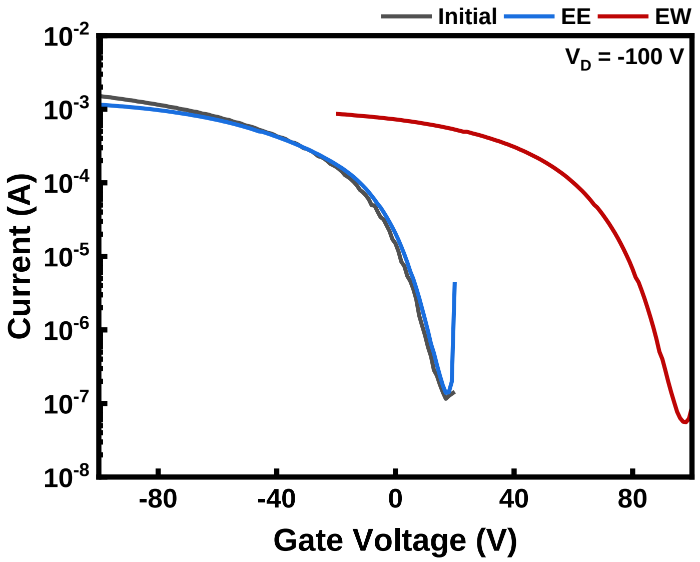

Figure S15. Transfer characteristics of the transistor memory comprising *s*-SWNT/PNDI-2T at initial (black), electrical writing (EW, V_g = 100 V for 1 s, red), and electrical erasing (EE, V_g = –100 V for 1 s, blue) states. Note that the measurements were conducted at V_d = –100 V.

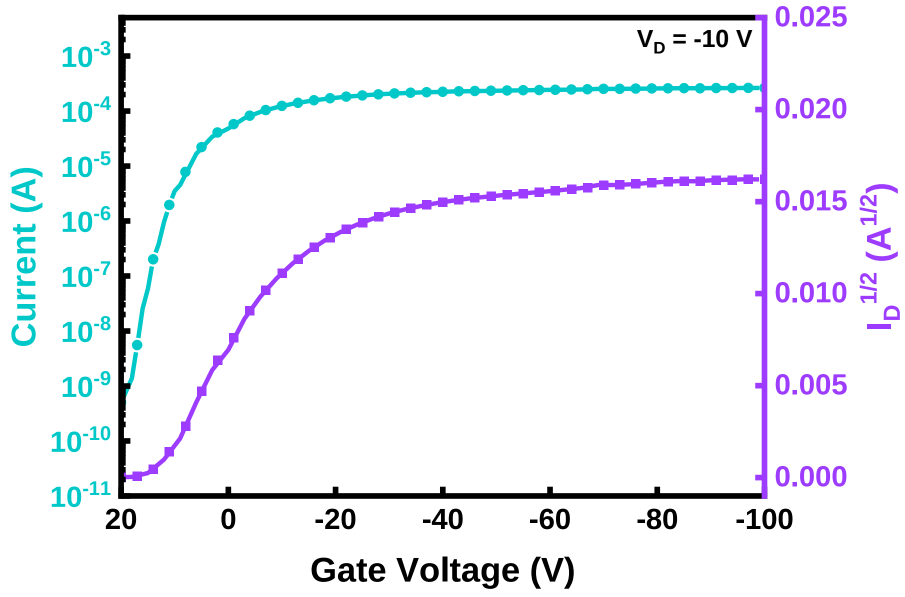

Figure S16. Transfer characteristics of the reference device comprising *s*-SWNT with V_D = –10 V and V_G swept from 20 to –100 V. The calculated $\mu_h = 5.34$ cm^2 V^{-1} s^{-1}.

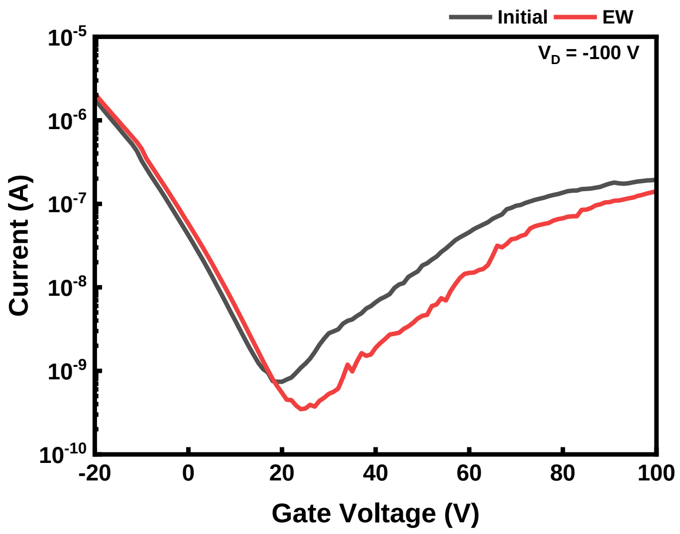

Figure S17. Transfer characteristics of the reference device comprising an N-type semiconductor of PNDI-2T at the initial (black), electrical writing (EW, V_g = 100 V for 1 s, red) states. Note that the measurements were conducted at V_d = –100 V.

Table S1. Memory device parameters of *s*-SWNT/PNDI-2T including the hole mobility, threshold voltage, and memory window/ratio at ON-state (electrical writing; V_g = +100 V; 1 s) and OFF-state (photoerasing; 254 nm; 80 s), and measured at different V_d of -10 or -100 V.

***s*-SWNT/PNDI-2T**	μ_h ($cm^2\ V^{-1}\ s^{-1}$)	$V_{th, writing}$ (V)	$V_{th, erasing}$ (V)	ΔV_{th} (V)	I_{on}/I_{off}
**V_d = -10 V**	0.50	83.6	7.9	75.7	10^5
**V_d = -100 V**	2.18	86.6	12.2	74.4	10^5

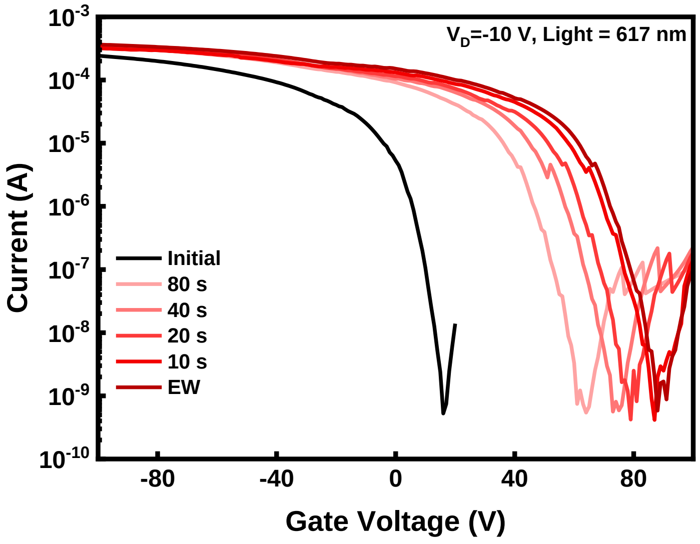

Figure S18. Transfer characteristics of the phototransistor memory with light-driven erasing (617 nm; 1 mW cm^{-2}) spanning the range of 10 to 80 s. Note that the measurements were conducted at V_d = –10 V.

Table S2. Memory device parameters of *s*-SWNT/PNDI-2T including the hole mobility, threshold voltage, and memory window/ratio at ON-state (electrical writing; V_g = +100 V; 1 s) and OFF-state (photoerasing; 254 or 617 nm; 80 s), and measured at V_d = –10 V.

Light wavelength/intensity	μ_h ($cm^2 V^{-1} s^{-1}$)	$V_{th, writing}$ (V)	$V_{th, erasing}$ (V)	ΔV_{th} (V)	I_{on}/I_{off}
**254 nm; 0.1 mW cm^{-2}**	0.50	83.6	7.9	75.7	10^5
**617 nm; 1 mW cm^{-2}**		81.4	52.0	29.4	2

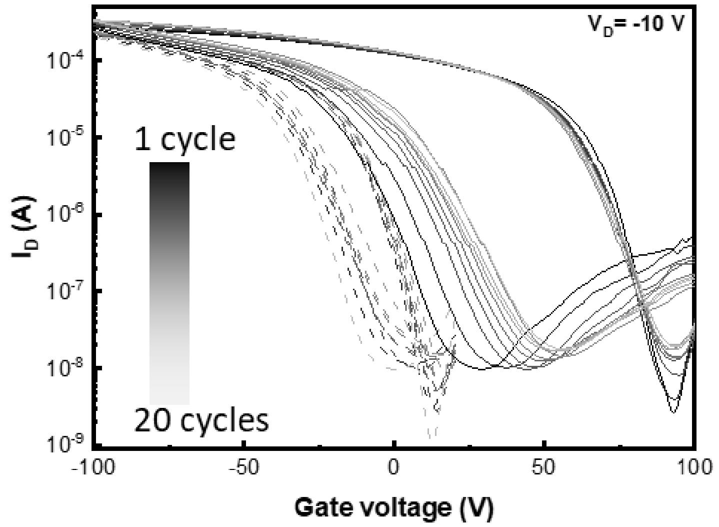

Figure S19. Transfer characteristics of the phototransistor memory with consecutive electrical writing (V_g = 100 V; 1 s; solid lines) and light-driven erasing (254 nm; 0.1 mW cm^{-2}; dashed lines). Note that the measurements were conducted at V_d = –10 V.

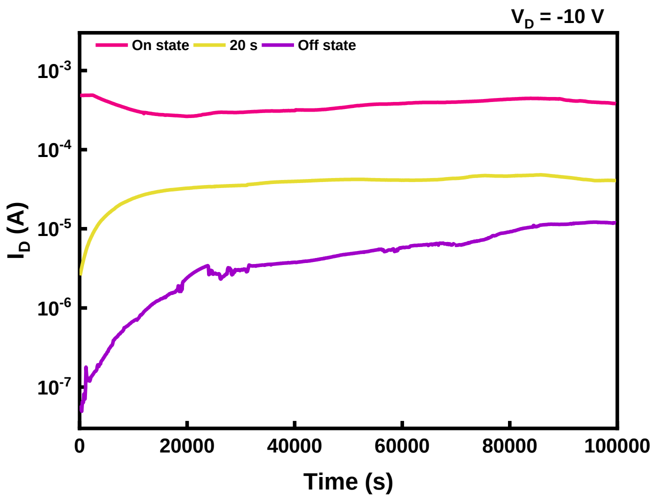

Figure S20. Long-term retention test of the memory device with electrical writing (ON state; V_g = 100 V; 1 s) and photoerasing (OFF state; 254 nm; 0.1 mW cm^{-2}; 20 or 80 s) in the duration of 100, 000 s.
